# E2F1 mediated DDX11 transcriptional activation promotes hepatocellular carcinoma progression through PI3K/AKT/mTOR pathway

**DOI:** 10.1038/s41419-020-2478-0

**Published:** 2020-04-24

**Authors:** Yan Yu, Dan Zhao, Kongfei Li, Yubo Cai, Penglin Xu, Rui Li, Juan Li, Xiaolong Chen, Ping Chen, Guangying Cui

**Affiliations:** 1 Precision Medicine Center, the First Affiliated Hospital of Zhengzhou University, Zhengzhou, 450052 China; 2 Key Laboratory of Clinical Medicine, the First Affiliated Hospital of Zhengzhou University, Zhengzhou, 450052 China; 3 Department of Oncology, The Third People’s Hospital of Zhengzhou, Zhengzhou, 450000 China; 4 Department of Hematology, Yinzhou People’s Hospital affiliated to Medical College of Ningbo University, Ningbo, 315000 China; 5 Zhejiang University School of Medicine, Hangzhou, 310058 China; 6 Nursing Department, The Third People’s Hospital of Zhengzhou, Zhengzhou, 450000 China; 7 Department of Infectious Diseases, Shulan Hospital, Hangzhou, 310012 China; 8 Department of Infectious Diseases, The Third People’s Hospital of Zhengzhou, Zhengzhou, 450000 China

**Keywords:** Cancer, Liver cancer

## Abstract

The DEAD/DEAH box helicase 11 (DDX11) plays vital roles in regulating the initiation of DNA replication. However, its precise function and regulation in hepatocellular carcinoma (HCC) have never been reported yet. In the current study, we found that DDX11 was overexpressed in HCC tissues. High DDX11 expression was positively correlated with large tumor size, tumor multiplicity, late tumor-node-metastasis (TNM) stage and poor prognosis. Additional, gain-of-function and loss-of-function experimental results revealed that DDX11 overexpression promoted HCC cell proliferation, migration, invasion and inhibited cell apoptosis in vitro. Overexpression of DDX11 also enhanced HCC tumorigenicity in vivo. Furthermore, DDX11 was transcriptionally regulated by transcription factor E2F1 in HCC, as demonstrated by chromatin immunoprecipitation (Ch-IP) and luciferase reporter assays. Mechanistically, E2F1/DDX11 axis promoted HCC cell proliferation, migration and invasion, at least in part, through activating PI3K/AKT/mTOR signaling pathway. Conclusively, our study demonstrates that E2F1-enhanced DDX11 expression promotes HCC progression through PI3K/AKT/mTOR pathway and DDX11 might be a potential therapeutic and prognostic target for HCC treatment.

## Introduction

Hepatocellular carcinoma (HCC), a primary liver malignancy, is the third leading cause of cancer-related mortality in human beings^1,2^. Patients are often diagnosed at late stages when curative treatments are not feasible, followed by low resectability rate, high recurrence and poor response to treatment, which contribute to the poor prognosis of HCC patients^3–5^. Therefore, novel biomarkers and new treatment targets to control HCC metastasis and recurrence are urgently needed.

Faithful DNA replication and proper sister chromatic cohesion ensure the correct propagation of the genetic material to daughter cells during cell division^6^. The DEAD/DEAH box helicase 11 (DDX11), a member of the DEAD/DEAH box family of helicases, was reported to be indispensable for chromosome arms’ cohesion. Mitotic failure occurred when it was depleted as replicated chromosomes were unable to segregate after prometaphase arrest^7^. DDX11 has been reported to share sequence similarity to the Fe-S cluster-containing DNA helicases FANCJ and RTEL1 that are vital in genome stability maintenance and its function was implicated in rare genetic syndromes and cancer development^8–12^. For example, autosomal recessive mutations of the DDX11-encoding gene were responsible for a rare cohesinopathy related disease named Warsaw breakage syndrome (WABS)^13^. Recently, high DDX11 expression was reported in melanomas, while suppressing DDX11 expression resulted in the inhibition of melanoma cell proliferation and cell death^14^. These results indicated that DDX11 might exert a critical role in tumorigenesis. However, the expression pattern, functional role and molecular mechanisms of DDX11 in HCC are largely unknown.

Numerous signaling pathways have been reported to be involved in progression and invasion of human tumors, such as the phosphoinositide 3-kinase (PI3K)/protein kinase B (AKT)/mechanistic target of rapamycin (mTOR) pathway^15^, Wnt/β-catenin signaling pathway^16^ and MAPK signaling pathway^17^. Meanwhile, among these signaling pathways, PI3K/AKT/mTOR signaling is frequently over-stimulated and influences the proliferation, metastasis and cell apoptosis in various types of cancers^18^, including gastric cancer^19^, breast cancer^20^, HCC^21–23^ and so on. Whether DDX11 regulates PI3K/AKT/mTOR signaling pathway in HCC development needs to be further clarified.

In this study, we reported that DDX11 was highly expressed and positively correlated with advanced TNM stage and poor survival in HCC. Function studies demonstrated that DDX11 served as an oncogene in HCC both in vitro and in vivo. Moreover, we revealed that DDX11 promoted HCC tumorigenesis through activation of the PI3K/AKT/mTOR pathway. In addition, we identified DDX11 as a novel transcriptional target of E2F1. We herein demonstrate that E2F1-regulated DDX11 serves as a crucial regulator in HCC development by regulating the activation of PI3K/AKT/mTOR pathway.

## Results

### DDX11 is upregulated in HCC tissues and associated with poor prognosis

**Fig. 1:**
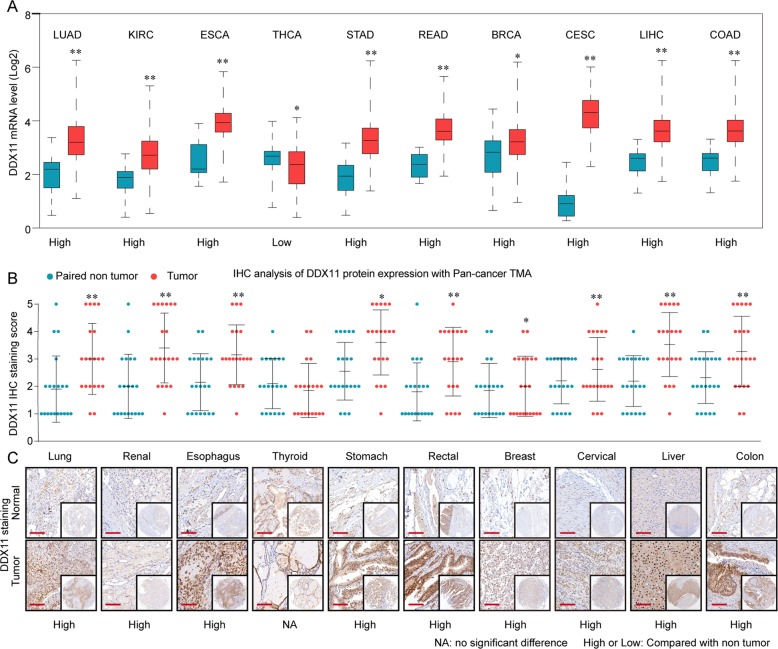
DDX11 is frequently upregulated in human solid tumors. **a** The mRNA expression of DDX11 in various tumor tissues and paired non-tumor tissues was analyzed based on TCGA database. Abbreviations: LUAD, lung cancer; KIRC, renal cancer; ESCA, esophagus cancer; THCA, thyroid cancer; STAD, stomach cancer; READ, rectal cancer; BRCA, breast cancer; CESC, cervical cancer; LIHC, liver cancer; COAD, colon cancer. **b** The protein expression of DDX11 in pan-cancer tissues and paired non-tumor tissues was analyzed by tissue microarray (TMA). **c** Representative IHC staining patterns of DDX11 in pan-cancer tissues and paired normal tissues. *p < 0.05, **p < 0.01, ***p < 0.001.

**Fig. 2:**
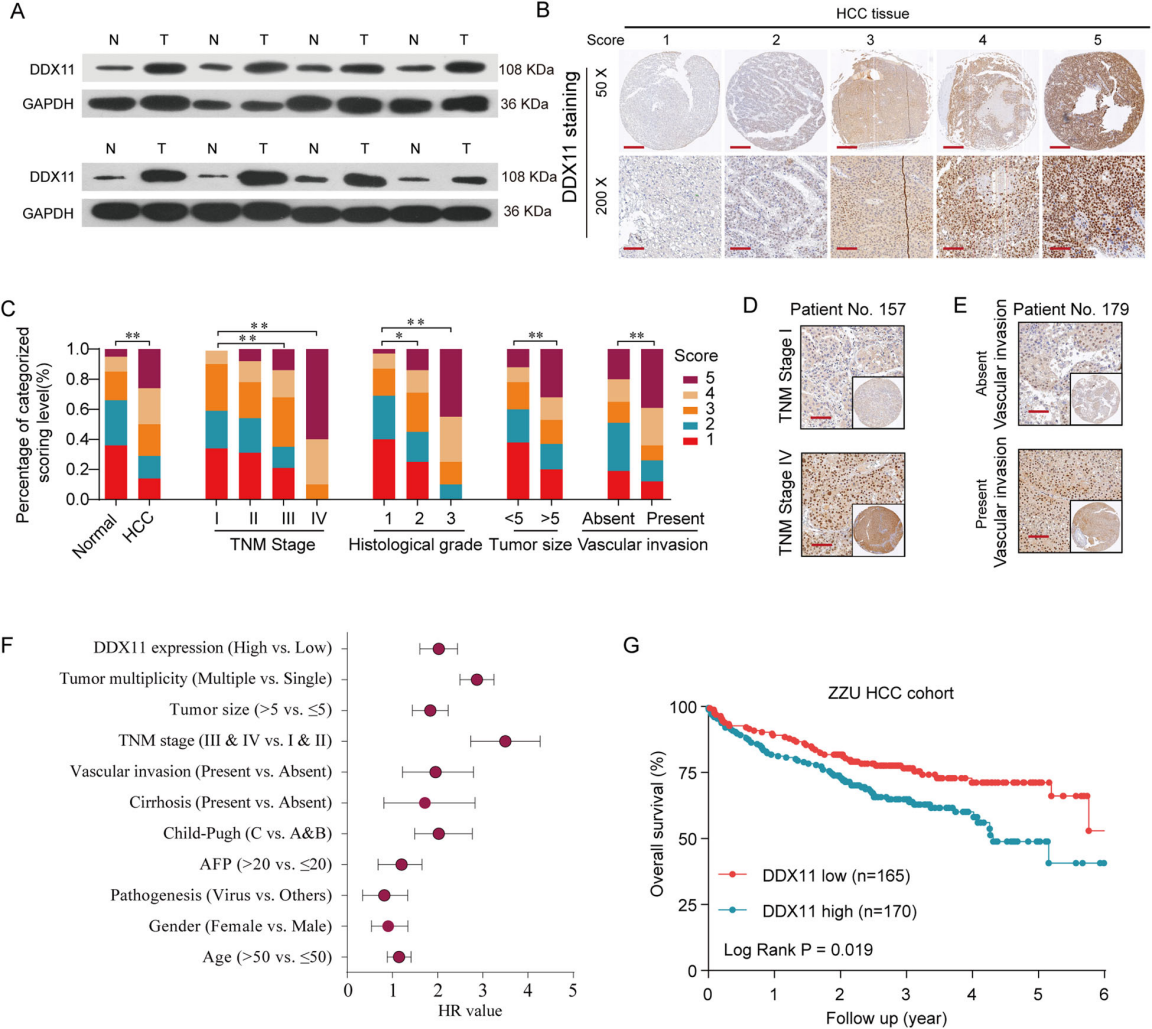
DDX11 is upregulated in HCC tissues and associated with poor prognosis. **a** Protein levels of DDX11 in 8 paired HCC and matched adjacent non-tumor tissues were determined by western blot assay. (N, matched adjacent non-tumor tissues; T, tumor tissues). **b** Representative expression pattern of DDX11 in HCC tissue samples. **c** Distribution of DDX11 immunohistochemical staining scores in HCC tissue samples according to TNM stage, histological grade, tumor size or vascular invasion. **d**, **e** Representative expression pattern of DDX11 in HCC tissue samples from patients with different TNM stages or in HCC tissue samples from patient with absent/present vascular invasion. **f** Univariate analysis of the association between DDX11 expression and clinicopathological features. **g** Kaplan–Meier analysis of the overall survival (OS) rate in HCC patients with high- or low- DDX11 expression in ZZU HCC cohort. *p < 0.05, **p < 0.01, ***p < 0.001.

To explore the expression pattern of DDX11 in HCC, we first analyzed the DDX11 expression level both in mRNA levels based on TCGA (The Cancer Genome Atlas) datasets, and in protein levels based on Pan-cancer tissue microarray (TMA). The results indicated that the DDX11 was frequently upregulated in multiple solid cancers, especially in HCC (Fig. 1a–c and Supplementary Figs. 1 and 2). To further confirm the expression levels of DDX11 in HCC, we examined the DDX11 protein expression in 8 paired HCC and adjacent non-tumor specimens. The results shown that 7/8 of HCC tissues had higher DDX11 expression (Fig. 2a). To determine the phenotypic expression of DDX11 protein in HCC clinical samples, IHC analysis was performed using a tissues microarray containing 396 pairs of HCC patient specimens and their corresponding adjacent normal tissues (ZZU HCC TMA cohorts, Supplementary Fig. 1D–J). We scored the DDX11 expression level (ranged from 1 to 5) based on the DDX IHC staining intensity (Fig. 2b) and found that the protein levels of DDX11 were remarkably higher in HCC tissues than those in the noncancerous counterparts (Fig. 2c). Intriguingly, clinic pathological analysis indicated that high DDX11 expression was positively related to advanced TNM stage, poor histological differentiation, larger tumor size, and high vascular invasion potential (Fig. 2c–e and Table 1). Additional, univariate Cox regression analysis indicated that high DDX11 expression was a potent independent risk indicator for survival in HCC patients (Fig. 2f). Kaplan–Meier analysis revealed that patients in the high-DDX11 group had a significant poor overall survival (OS) rate (Fig. 2g). Moreover, gene set enrichment analysis (GSEA) of the TCGA database addressed a high expression of DDX11 correlated with gene signatures of poor survival and high recurrence rate (Supplementary Fig. 3A, B). Meanwhile, univariate and multivariate analysis showed that DDX11 expression was an independent prognostic factor of OS, besides TNM stage (Table 2).

**Table 1:** Correlation of clinico-pathological features with DDX11 expression in ZZU HCC TMA cohort.

Variables	Clinicopathological features	DDX11 expression		*P* value
		Low expression (*n* = 165)	High expression (*n* = 170)	
Age (years)	≤53	88(53.3)	94(55.3)	0.718
	>53	77(46.7)	76(44.7)	
Gender	Male	121(73.3)	115(67.6)	0.254
	Female	44(26.7)	55(32.4)	
Pathogenesis	Virus	124(75.2)	131(77.1)	0.407
	Alcohol	25(15.2)	29(17.1)	
	Others	16(9.7)	10(5.9)	
Cirrhosis	Absent	22(13.3)	34(20.0)	0.102
	Present	143(86.7)	136(80.0)	
Vascular invasion	Absent	97(66.7)	119(62.4)	**0.032**
	Present	68(33.3)	51(37.6)	
TNM stage	Stage I and II	112(67.9)	96(56.5)	**0.031**
	Stage III and IV	53(32.1)	74(43.5)	
Tumor size(cm)	≤5	108(65.5)	82(48.2)	**0.001**
	>5	57(34.5)	88(51.8)	
Lymph metastasis	No	97(58.9)	91(53.5)	0.332
	Yes	68(41.2)	79(46.5)	
AFP	≤ 20	83(50.3)	80(47.1)	0.552
	>20	82(49.7)	90(52.9)	
Child-Pugh	A	19(11.5)	27(15.9)	0.777
	B	76(46.1)	80(47.1)	
	C	70(42.4)	63(37.1)	
Tumor multiplicity	Single	75(45.5)	81(47.6)	0.687
	Multiple	90(54.5)	89(52.4)	
DDX11 expression	Live	122(73.9)	105(61.7)	**0.017**
	Dead	43(26.1)	65(38.2)	

Bold values indicate statistical significance, *P* < 0.05.

**Table 2:** Independent prognostic factors for OS by multivariate analyses in ZZU HCC TMA cohort.

Univariate analysis	Relative risk	95% CI	*P* value
TNM stage (stage III/IV vs. stage I/II)	2.823	1.915-4.203	**0.006**
Vascular invasion (present vs. absent)	1.779	1.118-2.392	**0.039**
Tumor size (>5 cm vs. ≤5 cm)	1.232	0.924-1.817	**0.064**
DDX11 expression (high vs. low)	1.997	1.334-3.346	**0.028**

Bold values indicate statistical significance, *P* < 0.05.

**Fig. 3:**
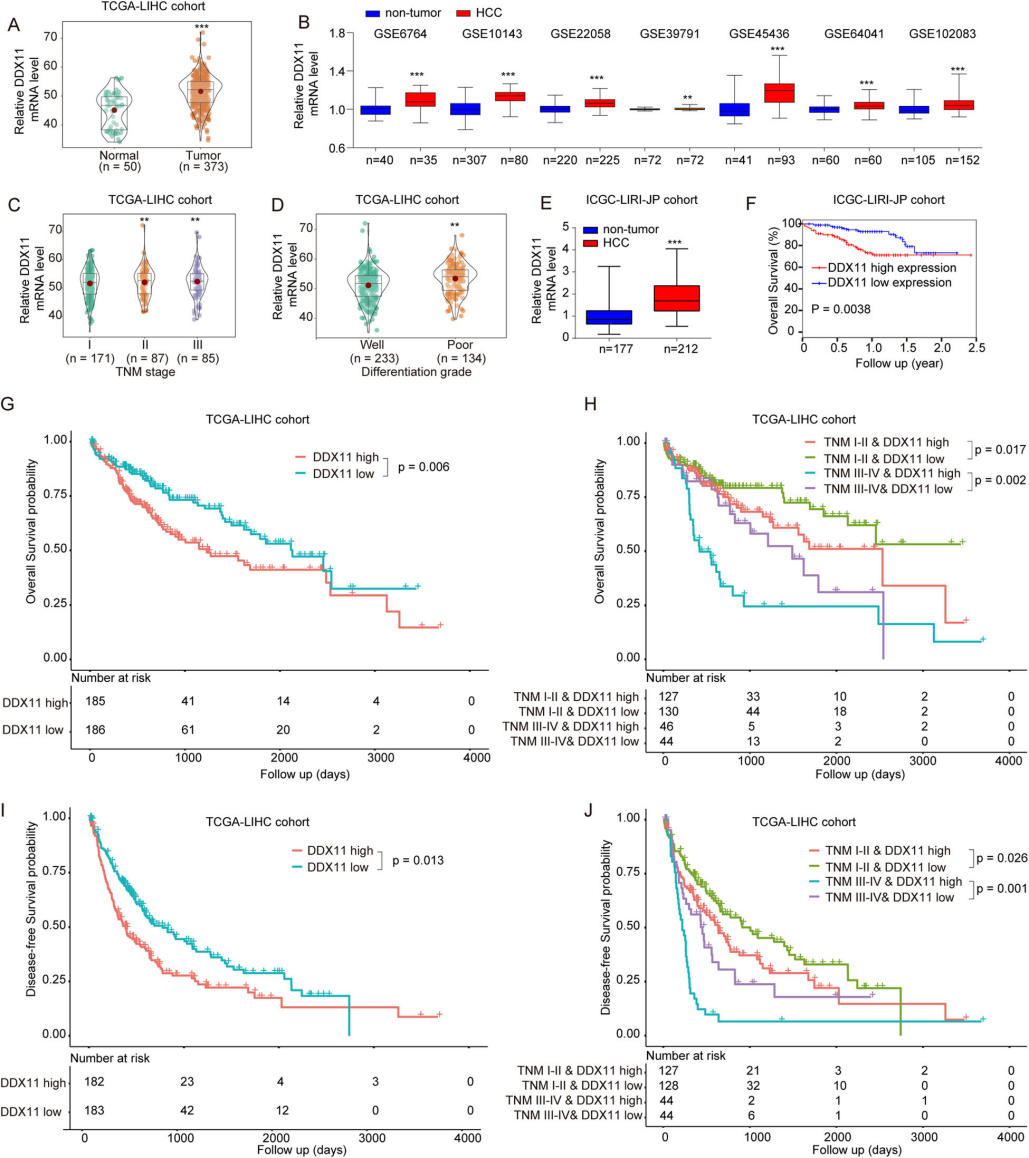
Bioinformatics analysis of DDX11 expression in TCGA and GEO database. **a**, **b** DDX11 mRNA expression levels were analyzed in HCC tissues from TCGA-LIHC cohort or GEO databases. **c**, **d** DDX11 mRNA expression levels were analyzed in HCC patients with different TNM stage or differentiation grade based on the dataset from TCGA-LIHC cohort. **e**, **f** DDX11 mRNA expression in HCC tissues or non-tumor control tissues and the correlation between OS and high- or low- DDX11 expression were in ICGC-LIRI-JP cohort. **g**, **h** OS and DFS analysis of the HCC patients with high- or low- DDX11 expression in TCGA-LIHC cohort. **i**, **j** OS and DFS analysis of the HCC patients with different TNM stages and DDX11 expression. *p < 0.05, **p < 0.01, ***p < 0.001.

Furthermore, from TCGA and GEO (Gene Expression Omnibus) database, we also validated that DDX11 was highly expressed in HCC tissues and positively associated with late TNM stage and poor differentiation grade (Fig. 3a–d). Similar results were also obtained in the ICGC-LIRI-JP cohort (Fig. 3e and f). There was also a significant positive relationship between DDX11 expression and AFP/Ki-67 levels (Supplementary Fig. 4A). Detailed analysis revealed that both in early TNM stages (stage I and II) and late stages (stage III and IV)), patients with high DDX11 expression had shorter OS and disease-free survival (DFS) durations than patients with low DDX11 expression in TCGA-LIHC cohorts, which indicated that DDX11 expression level might be indicative of the prognosis of HCC patients at various clinical stages (Fig. 3g–j). In addition, there was no significant difference of DDX11 expression between cirrhotic tissue and normal tissue, indicating DDX11 overexpression might be a typical feature of HCC (Supplementary Fig. 4B). Moreover, the area under the ROC curve (AUC) of DDX11 in distinguishing normal liver tissues and HCC tissues was 0.849 (Supplementary Fig. 4C). Taken these results together, DDX11 could be a novel prognostic and diagnostic biomarker for HCC patients.

### Knockdown of DDX11 suppresses cell proliferation, migration and invasion, and induces apoptosis of HCC cells in vitro

**Fig. 4:**
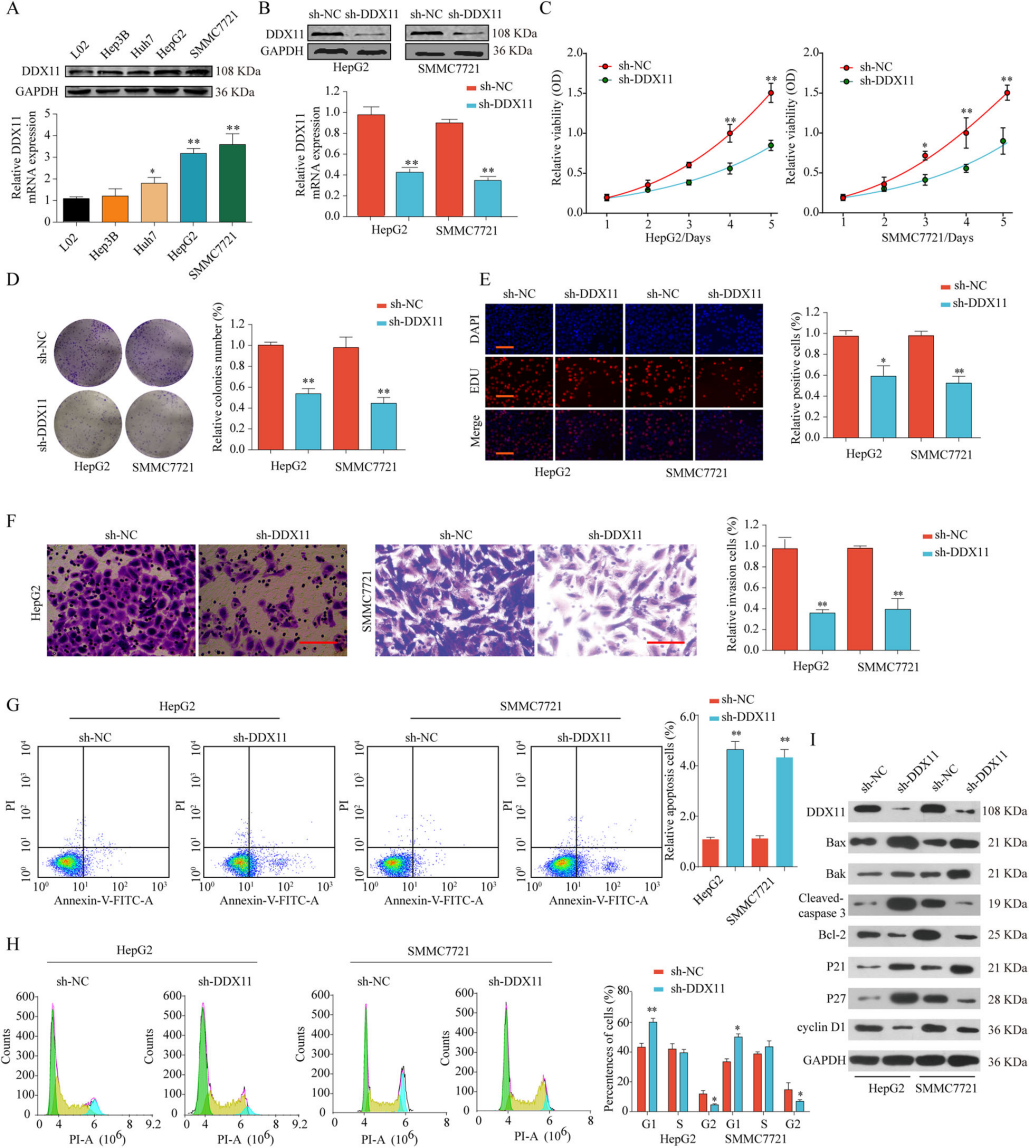
Knockdown of DDX11 suppresses cell proliferation, migration and invasion, and induces apoptosis of HCC cells in vitro. **a** DDX11 protein and mRNA expression in HCC cell lines or in normal liver cell line were analyzed by western blot and qPCR. **b** Western blot and qPCR analysis of DDX11 expression in HepG2 or SMMC7721 cells after transfection of sh-NC or sh-DDX11. HepG2 or SMMC7721 cells were transfected with sh-NC or sh-DDX11. **c**–**e** Cell proliferation and DNA synthesis were analyzed by CCK-8 assay, colony formation assay and EdU immunofluorescence staining respectively. **f** Cell invasion ability was determined by transwell assay. **g**, **h** Cell apoptosis and cell cycle analysis of HepG2 or SMMC7721 cells transfected with sh-NC or sh-DDX11 were analyzed by flow cytometer using Annexin V/PI or PI staining. **i** Western blot analysis of cell apoptosis and cell cycle related proteins in HepG2 or SMMC7721 cells with or without DDX11 knockdown. *p < 0.05, **p < 0.01, ***p < 0.001.

Next, we investigated the biological function of DDX11 in vitro. As shown in Fig. 4a, DDX11 expression was significantly enhanced in HCC cell lines, especially in SMMC7721 and HepG2 cells compared with normal liver cell line L02. We constructed DDX11 stable knockdown HepG2 and SMMC7721 cell lines using lentiviral-based approach. The knockdown efficiency was confirmed by western blot and qPCR (Fig. 4b). CCK-8, colony formation, and EdU assays showed that DDX11 silencing suppressed the cells proliferation, colony formation and DNA synthesis (Fig. 4c–e). The capabilities of cell invasion and migration of HepG2 or SMMC7721 cells were significantly decreased after DDX11 knockdown as shown by transwell and wound-healing assays (Fig. 4f and Supplementary Fig. 4D). Moreover, we found dramatically increased apoptotic cells in sh-DDX11 group compared with that in sh-NC group (Fig. 4g). The proportion of G2 phase in HepG2 or SMMC7721 cells was also decreased after DDX11 knockdown (Fig. 4h). Furthermore, we observed a decrease in the anti-apoptotic proteins (Bcl-2, cyclin D1), and an increase in the pro-apoptotic proteins (Bax, Bak, and P21) in cells transfected with the lentivirus silencing DDX11 (Fig. 4i). These findings suggested that loss of DDX11 suppressed cell proliferation, migration, invasion, and induced apoptosis in HCC cells.

### Overexpression of DDX11 promotes cell proliferation, migration and invasion, and inhibits apoptosis of HCC cells in vitro

To further address the role of DDX11 in HCC progression, we employed the gain-of-function strategy and both protein and mRNA levels of DDX11 were markedly increased in Hep3B or Huh7 cells after transfecting with pcDNA-DDX11 (oe-DDX11) (Supplementary Fig. 5A). Functionally, we found that DDX11 overexpression enhanced the proliferative potential of Hep3B or Huh7 cells, as demonstrated by CCK-8 assay, colony formation assay, and EdU assay (Supplementary 5B–D). The migratory and invasive ability was also enhanced in cells transfected with oe-DDX11 (Supplementary Fig. 5E, F). Furthermore, the apoptotic cells were decreased while the proportion of G2 phase was increased after DDX11 overexpression in Hep3B or Huh7 cells (Supplementary Fig. 5G, H). The loss-of-function results combined with the gain-of-function data revealed that DDX11 facilitated HCC growth and invasion.

### DDX11 promotes HCC development in vivo

**Fig. 5:**
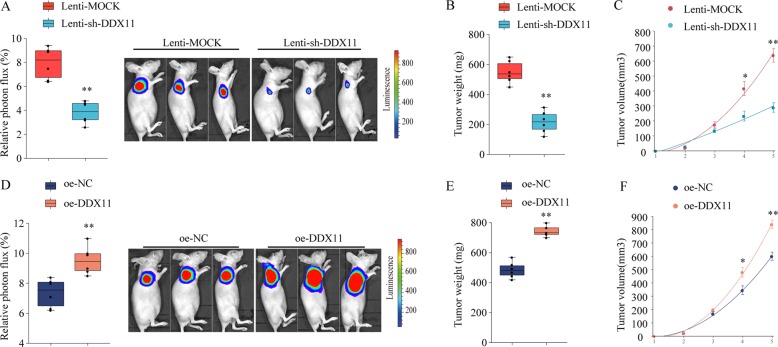
DDX11 promotes HCC progression in vivo. SMMC7721 cells infected by lentiviral to achieve stable knockdown (Lenti-sh-DDX11) or overexpression of DDX11 (oe-DDX11) or infected by negative control (Lenti-MOCK or oe-NC, respective) were implanted into the nude mice and tumor growth was recorded. **a** The relative luciferase activity and the representative photon flux of HCC tumor in nude mice from Lenti-Mock or Lenti-sh-DDX11 group were determined using a live imaging system to measure the luciferase signal. **b** Tumor weight in nude mice of Lenti-Mock or Lenti-sh-DDX11 group was assessed at day 35. **c** Tumor volume (growth curves of tumor) was determined based on tumor size measured every week. **d** The relative luciferase activity and the representative photon flux of HCC tumor in nude mice from oe-NC or oe-DDX11 group were determined using a live imaging system to measure the luciferase signal. **e** Tumor weight in nude mice of oe-NC or oe-DDX11 group was assessed at day 35. **f** Tumor volume (growth curves of tumor) was determined based on tumor size measured every week. *p < 0.05, **p < 0.01, ***p < 0.001.

To further validate the function of DDX11 in vivo, HCC xenograft tumor model in athymic nude mice were established to evaluate the effect of DDX11 knockdown or overexpression in vivo. The athymic nude mice were subcutaneous injected with Hep3B cells with stable DDX11 knockdown (sh-DDX11)/overexpression (oe-DDX11) or with negative control (sh-NC, oe-NC, respective). As shown in Fig. 5a–d, knockdown DDX11 inhibited HCC tumor growth, with significantly lower tumor weight and smaller tumor volume. In contrast, in mice inoculated with Hep3B-DDX11-overexpressing cells, tumors developed faster than that in mice inoculated with control Hep3B cells, with markedly high tumor weight and larger tumor volume (Fig. 5e–h). These findings suggested that DDX11 promoted HCC growth in vivo.

### DDX11 promotes HCC growth by activating PI3K/AKT/mTOR pathway

**Fig. 6:**
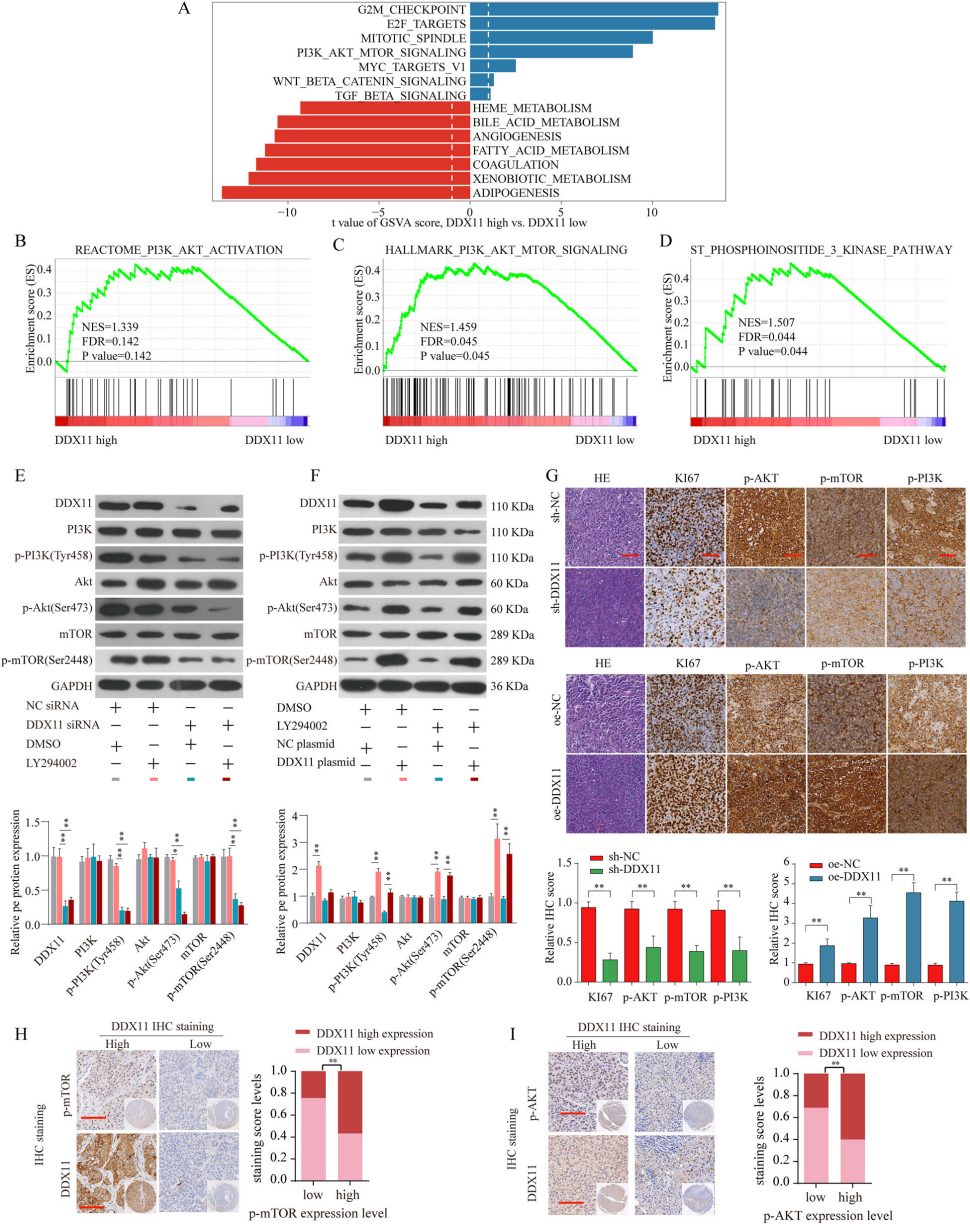
DDX11 promotes HCC growth by activating PI3K/AKT/mTOR pathway. **a** GSVA analysis showed that PI3K/AKT/mTOR pathway in tumor with DDX11-high expression compared with those with DDX11-low expression was identified as the top three activated regulated pathways in TCGA HCC cohort. **b**–**d** GSEA analysis the enrichment of the PI3K/AKT/mTOR signaling pathway genes with different DDX11 expression. **e** Expression levels of PI3K, p-PI3K(Tyr458), Akt, p-Akt (Ser473), mTOR, and p-mTOR (Ser2448) in SMMC7721 transfected with NC siRNA & DMSO (gray), NC siRNA & LY294002 (pink), DDX11 siRNA & DMSO (green) or DDX11 siRNA & LY294002 (brown) were analyzed by western blot. **f** Expression levels of PI3K, p-PI3K(Tyr458), Akt, p-Akt (Ser473), mTOR, and p-mTOR (Ser2448) in SMMC7721 transfected with NC plasmid & DMSO (gray), DDX11 plasmid & DMSO (pink), NC plasmid & LY294002 (green) or DDX11 plasmid & LY294002 (brown) were analyzed by western blot. The representative result of at least three independent experiments was shown. **g** IHC staining of Ki-67, p-PI3K, p-AKT, and p-mTOR in tumor tissues from subcutaneous transplanted tumor model. **h**–**i** Representative IHC staining of DDX11 and p-mTOR or p-AKT in HCC tissues from ZZU cohort and quantification of DDX11 staining scores in HCC patients from ZZU cohort with different p-mTOR or p-AKT expression. Scale bars, 50 μm. *p < 0.05, **p < 0.01, ***p < 0.001.

To explore the underlying mechanisms of DDX11 in HCC progression, comprehensive bioinformatics analysis was performed and results exhibited a positive correlation between highly DDX11 expression and abnormally activated cell cycle, DNA replication, homologous recombination, and mismatch repair pathway (Supplementary Fig. 6A–E). Of these, PI3K/AKT/mTOR pathway was an overwhelmingly enriched gene set in DDX11 high expression HCC tissues (Supplementary Fig. 6C, Fig. 6a–d). We further confirmed that the expression of PI3K/AKT/mTOR pathway proteins, such as phosphorylated-PI3K (p-PI3K), phosphorylated-AKT (p-AKT) and mTOR was decreased after DDX11 knockdown, whereas increased with DDX11 overexpression (Fig. 6e, f). Furthermore, LY294002 (a PI3K signaling inhibitor) was used to inhibit the PI3K/Akt/mTOR pathway and the results revealed that DDX11 silencing could further enhance the inhibitory effect of LY294002 while DDX11 overexpression could reverse the inhibitory effect of LY294002 in HCC cells (Fig. 6e, f).

Moreover, we examined the expression of p-PI3K, p-AKT, and mTOR in xenograft tumors by IHC staining. Consistent with the western blotting results, we demonstrated that p-PI3K, p-AKT, and p-mTOR were downregulated in the xenograft tumors with DDX11 knockdown; in contrast, p-PI3K, p-AKT, and p-mTOR were enhanced in xenograft tumors injected with DDX11 overexpressing Hep3B cells (Fig. 6g). Moreover, the expression of proliferation biomarker Ki-67 was decreased in the DDX11-knockdown group, whereas increased in DDX11-overexpression group (Fig. 6g). Meanwhile, IHC staining of DDX11 was positively associated with the activation of mTOR and AKT expression in HCC tissues (Fig. 6h, i). These results corresponded to the proliferation and invasion abilities of HepG2 cells (Supplementary Fig. 7A, B). Collectively, these data further strengthened the conclusion that DDX11 promoted HCC progression by activating the PI3K/AKT/mTOR pathway.

### E2F1 activates DDX11 transcription in HCC cells

**Fig. 7:**
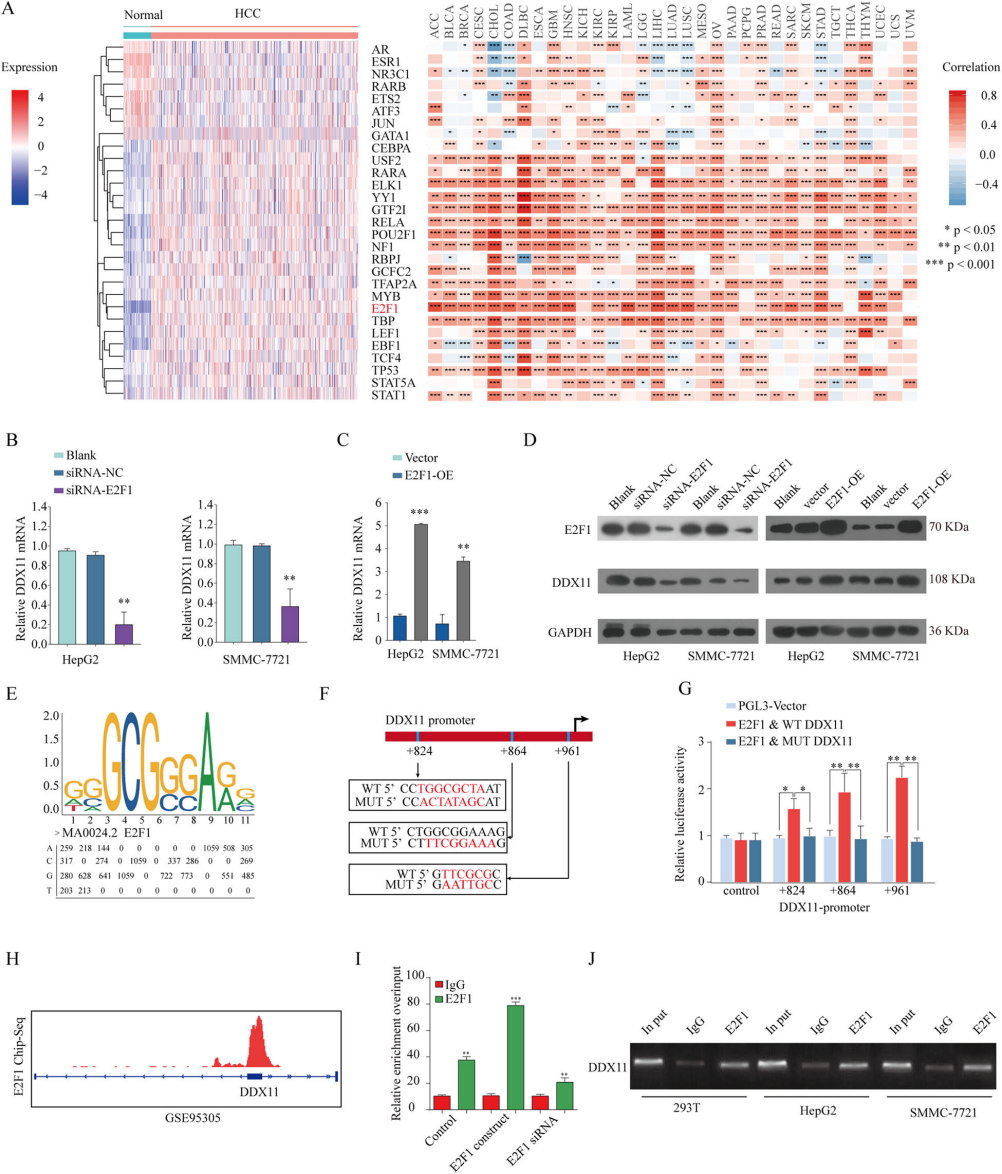
E2F1 activates DDX11 transcription in HCC cells. **a** E2F1 was speculated as an upstream factor of DDX11 based on differential E2F1expression analysis between normal tissues and tumor tissues (**a** left panel) and Pearson analysis of the correlation between DDX11 and E2F1 expression in TCGA-LIHC cohort (**a** right panel). **b**, **c** qPCR and western blot (**d**) analysis of the E2F1 and DDX11 expression in HepG2 or SMMC7721 cells transfected with E2F1 plasmid and E2F1 siRNA, respectively. **e** The sequence logo of a potential E2F1 binding site in JASPAR and a diagram of mutant sites in the DDX11 sequence. **f**, **g** Dual-luciferase reporter assay was performed by co-transfection of the DDX11 promoter fragment (WT or MUT pGL3-DDX11) and an E2F1-overexpression construct into HEK293 cells. **h** ChIP-seq analysis of E2F1 binding in DDX11 gene locus based on the data from GSE95305. **i**, **j** ChIP assay was performed using E2F1 antibody to determine the E2F1 binding to the DDX11 gene promoter. *p < 0.05, **p < 0.01, ***p < 0.001.

Previous studies have identified that transcriptional factors exert critical roles in the progression of human neoplasm^24,25^. To screen for the potential transcription factors regulating DDX11 expression, we predicted 29 potential transcription factor candidates that regulate DDX11 expression using PROMO software (http://alggen.lsi.upc.es/cgi-bin/promo_v3)^26^ and performed difference analysis based on TCGA LIHC database (Fig. 7a left panel). We also analyzed the relationship between E2F1 and DDX11 expression in TCGA database and found that E2F1 expression was positively correlated with DDX11 expression in these cancers. Especially, there was a significant positive relationship between DDX11 and E2F1 expression in HCC tissues (Fig. 7a right panel, Supplementary Fig. 8). Given the essential role of E2F1 in the HCC progression^27^, E2F1 was speculated as the upstream transcription factor of DDX11. To confirm this speculation, validation experiments were performed. We demonstrated that DDX11 expression was significantly enhanced after transfection with E2F1 construct, whereas decreased with E2F1 knockdown in HCC cells both in mRNA (Fig. 7b, c) and protein levels (Fig. 7d). To further identify whether E2F1 could regulate DDX11, the JASPAR (jaspar.genereg.net) database was used to analyze the potential E2F1 binding site in the DDX11 promoter sequence (Fig. 7e). We mutated the predicted E2F1 binding sites (+824, +864, +961) on DDX11 sequence and luciferase activity assay showed that DDX11 transcriptional activity was significantly increased after transfection with E2F1 overexpression plasmid, but the enhanced luciferase activity was reversed by transfection of the mutated DDX11 sequence (Fig. 7f, g).

Furthermore, E2F1 ChIP-seq data were downloaded from GEO (GSE95305) and analyzed, which showed the potential binding of E2F1 on DDX11 gene locus (Fig. 7h). Moreover, ChIP assay was performed to ascertain whether E2F1 directly bound to DDX11 in HCC. The results revealed that E2F1 could bind to the promoter region of DDX11 gene locus in HCC cells (Fig. 7i, j). In summary, E2F1 transcriptionally enhanced DDX11 expression by binding to its promoter region in HCC cells.

### E2F1/DDX11 axis promote HCC cell proliferation and invasion through activating PI3K/AKT/mTOR pathway

**Fig. 8:**
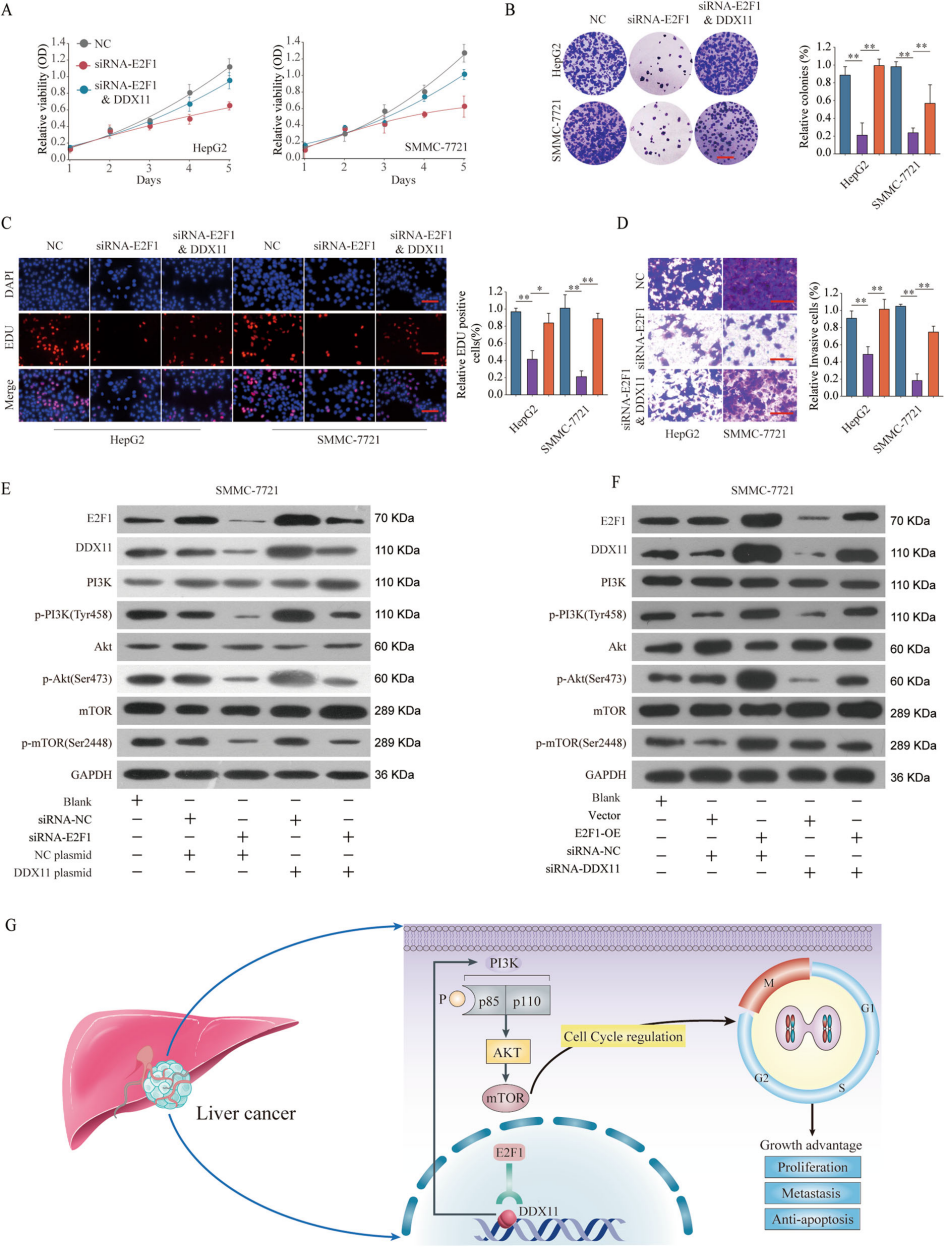
E2F1/DDX11 axis contributes to HCC cell proliferation and invasion through activating PI3K/AKT/mTOR pathway. HepG2 or SMMC7721 cells were transfected with negative control (NC), siRNA targeting E2F1 (siRNA-E2F1), or siRNA-E2F1 & DDX11 overexpression plasmid (DDX11). **a**–**c** Cell proliferation of HepG2 or HuH-6 cells was assessed by CCK-8 assay, colony formation assay or EDU staining assay. Scale bars, 50 μm. **d** Cell invasion ability of HepG2 or SMMC7721 cells in different groups was analyzed by transwell assay. Scale bars, 50 μm. **e** Expression levels of PI3K/p-PI3K(Tyr458), Akt/p-Akt (Ser473), and mTOR/p-mTOR (Ser2448) in SMMC7721 transfected with negative control, siRNA-E2F1, negative control plasmid, or DDX11 plasmid were analyzed by western blot. **f** Expression levels of PI3K/p-PI3K(Tyr458), Akt/p-Akt (Ser473), and mTOR/p-mTOR (Ser2448) in SMMC7721 transfected with negative control, E2F1 plasmid, negative control siRNA, or DDX11 siRNA were analyzed by western blot. The representative result of at least three independent experiments was shown. **g** Schematic representation showing E2F1/DDX11 axis mediated aggressive behaviors in HCC through activating PI3K/AKT/mTOR signaling pathway. *p < 0.05, **p < 0.01, ***p < 0.001.

To demonstrate the effects of E2F1/DDX11 axis on HCC cells, we conducted functional recused experiments. CCK-8, EDU staining, and colony formation experiments demonstrated that E2F1 knockdown suppressed proliferative potential in both HepG2 and SMMC-7721 cell lines but this suppression effect could be partially reversed by DDX11 overexpression (Fig. 8a–c). Meanwhile, DDX11 overexpression partially rescued the cell invasion of HepG2 or SMMC-7721 cells with E2F1 silencing (Fig. 8d). Additional, HCC patients with E2F1&DDX11 dual high expression trended toward correlation with worse survival rate compared with those with E2F1&DDX11 dual low expression (Supplementary Fig. 9A, B).

We further evaluated the function of E2F1/DDX11 axis on PI3K/Akt/mTOR signaling. The results suggested that E2F1 knockdown inhibited the activation of the PI3K/Akt/mTOR signaling and DDX11 overexpression could reverse the inhibitory effect (Fig. 8e). Meanwhile, DDX11 knockdown partially rescued the enhancement effect on PI3K/Akt/mTOR signaling in E2F1 overexpression group (Fig. 8f). Taken together, these findings demonstrate that E2F1 promotes HCC cell proliferation and invasion through enhancing DDX11 expression, which involves the PI3K/Akt/mTOR signaling pathway (Fig. 8g).

## Discussion

DDX11 has been identified to involve in the process of the lagging strand during DNA replication and in the maintenance of the fork structure for the establishment of cohesion^7^. Recently, DDX11 has been linked to the progression of multiple cancers, including melanoma and lung cancer^14,28^. However, its functional role and clinical significance in HCC have never been reported yet. In our present study, we primarily focused on the clinical significance and cell proliferation function of aberrant DDX11 expression, and demonstrated that E2F1-enhanced DDX11 expression could promote hepatocellular carcinoma progression through PI3K/AKT/mTOR pathway and DDX11 might be a potential therapeutic or prognostic target for HCC treatment.

Emerging evidence has shown that DDX11 is highly expressed and plays vital roles in the progression of multiple tumors. For instance, our previous study reported that the high expression level of DDX11 was associated with unpleasant prognosis of lung cancer^28^. Bhattacharya et al. reported that DDX11 enhanced melanoma proliferation and metastasis^29^. Consistent with these results, our data indicated that DDX11 was highly expressed in HCC tissues and cell lines, and upregulated DDX11 expression was associated with poor prognosis. Moreover, in vitro and in vivo functional studies indicated that knockdown of DDX11 expression significantly impeded cell growth. In addition, we found that DDX11 silencing also inhibited cell migration/invasion and promoted cell apoptosis, arrested cell cycle in G1/S phase, whereas ectopic DDX11 expression promoted these biological processes. These results validated that DDX11 played an important role in the progression of HCC.

We further investigated the underlying mechanisms of DDX11 in promoting HCC tumorigenesis. Bioinformatics analysis indicated that DDX11 expression was closely correlated with the expression of PI3K/AKT pathway gene sets. Numerous studies confirmed that the PI3K/AKT/mTOR pathway exerted an important influence on cell proliferation and growth, including HCC. For example, Wang et al. reported that hydrogen sulfide activated PI3K/AKT/mTOR pathway to promote cancer progression in HCC^30^. In esophageal squamous cell carcinoma, ricolinostat (ACY-1215) could suppressed proliferation and promotes apoptosis via PI3K/AKT/mTOR pathway^31^. Moreover, studies by Wang et al. demonstrated that PI3K/AKT/mTOR signaling pathway could promote autophagy of hepatocellular carcinoma cells regulated by Alpha-fetoprotein (AFP)^32^. In our study, we demonstrated that DDX11 promoted proliferation, invasion, and migration in HCC via activation of the PI3K/AKT/mTOR pathway. These results confirmed that DDX11 exerted its oncogenic role in HCC mainly through the activation of the PI3K/AKT/mTOR pathway.

To verify the potential transcription factors that affected the overexpression of DDX11 in HCC, we conducted bioinformatics analysis to predict the transcriptional factors that could regulate the expression of DDX11. The E2F-family members were served as candidates because they played crucial roles in controlling the cell cycle and action of tumor-related proteins^33,34^. We found that the expression of E2F1, a member of E2F protein family, was positively correlated with DDX11 mRNA expression in most cancer types. E2F1 is a transcription factor involved in the regulation of many cellular processes, such as cell proliferation, invasion, and apoptosis. It also plays important roles during the development of human malignancies, including HCC^35–37^. In this study, our data demonstrated that E2F1 bound to DDX11 promoter region and promoted DDX11 expression, leading to the activation of PI3K/AKT/mTOR signaling pathway. Mechanistically, E2F1 silencing suppressed the activation of the PI3K/AKT/mTOR signaling, while DDX11 overexpression could reverse the inhibitory effect caused by E2F1 loss-function. These results indicated that DDX11 might be a downstream effector of E2F1 that could control HCC cell migration and invasion.

In summary, our study demonstrates for the first time that DDX11 is an oncogene in HCC and DDX11 regulates HCC cell proliferation and invasion via activating PI3K/AKT/mTOR signaling pathway. Moreover, E2F1 could transcriptionally enhance DDX11 expression by binding to its promoter region in HCC cells. Taken together, DDX11 may be a promising prognosis biomarker and a potential therapeutic candidate for HCC treatment.

## Materials and methods

### Patients and specimens

The detailed information of the patients and specimens used in this study has been well described in our previous study^38,39^. Briefly, the Pan-cancer Tissue microarray (Pan-cancer TMA) containing 10 tumor types (each type of cancer containing 20 specimens and adjacent non-tumor tissues) were constructed with clinical specimens obtained between April 2009 and December 2012 from the First Affiliated Hospital of Zhengzhou University, Zhengzhou University, China. Moreover, 396 paired paraffin embedding HCC specimens (341 with available follow-up data) and corresponding non-tumor tissues obtained from the First Affiliated Hospital of Zhengzhou University from 2009 to 2012 (ZZU cohort) were constructed using the diameter of 1.5-mm cores. All the experiments were approved by the Institutional Review Board of the First Affiliated Hospital of Zhengzhou University.

### Cell culture and transfection

The human HCC cell lines (SMMC7721, Hep3B, HepG2, Huh7) and normal liver cell (L02) were purchased from ATCC (Manassas, USA) or Sibcb (Shanghai, China). Cells were cultured in accordance with their directions. DDX11 plasmids and shRNA, E2F1 plasmids and their negative controls (NC) were from Ambion (Austin, USA). All transfections were conducted with Lipofectamine3000 reagent (Invitrogen, Carlsbad, USA). Cells used in this study were listed in Supplementary Table 3.

### Gene set enrichment analysis (GSEA) and gene set variation analysis (GSVA)

GSEA was applied to analyze which gene sets were related to DDX11 expression in TCGA-LIHC database. The expression profiles of 377 samples from TCGA-LIHC dataset were grouped into two classes according to gene expression. GSEA v2.0 was then performed to verify whether the gene sets from the MSigDB database v4.0 were randomly distributed at the top or bottom of the ranking. The statistical significance threshold was set at *P* < 0.05.

### Collection of liver cancer microarray datasets

A sum of 375 liver cancer data and 50 non-tumor data, with at least 10 years of follow-up was collected for gene expression analysis and survival analysis from The Cancer Genome Atlas (TCGA, https://tcga-data.nci. nih.gov/tcga/, updated to the end of December 31, 2016) database.

Seven liver cancer mRNA microarray datasets accompanied with scientific publications were gathered and analyzed through the Gene Expression Omnibus (GEO) of the National Center for Biotechnology Information (NCBI). The BRB-array tools were performed to determine the differentially expressed genes between normal liver tissues and HCC samples in each dataset. The detailed information was showed in Supplementary Table 4.

### Quantitative real-time PCR (qPCR)

The detailed procedures were described in our previous study^40^. Briefly, total RNA was extracted from cultured cells using TRIzol reagent (Life Technologies, Carlsbad, USA) following the manufacturer’s protocol. QPCR was conducted with power up SYBR Green kit (ABI, Foster City, USA). The relative expression of the target genes was normalized to the β-actin. The data analyses were performed using the 2^−ΔΔCt^ method. The primer sequences were provided in Supplementary Table 5.

### Cell proliferation assays

The HCC cell viability was monitored using a Cell Counting Kit-8 (CCK-8) (Dojindo, Japan). Cells (1 × 10^3^/well) transfected with DDX11 plasmids or shRNA were seeded into a 96-well plate. At every other day until day 5, the absorbance values of each well were measured at 450 nm with a plate spectrophotometer (Molecular Devices, Sunnyvale, CA, USA) according to the manufacturer’s instructions. For colony formation assay, 5 × 10^3^ cells were plated in 6-well plates. After 2 weeks, the cells were fixed with 30% formaldehyde for 15 min and then stained with 0.1% crystal violet. The colonies (containing more than 50 cells) number was determined under an optical microscope. The DNA synthesized rate was assayed with 5-ethynyl-20-deoxyuridine (EdU) assay kit (Ribobio, Guangzhou, China). Images were photographed and analyzed with a microscope (Olympus, Tokyo, Japan). The ratio of EdU-stained cells (red fluorescence) to Hoechest-stained cells (blue fluorescence) was calculated to assess the cell proliferation activity.

### Wound healing assay and transwell invasion assay

SMMC7721 and HepG2 cells (5 × 10^6^) were seeded into 6-well plates after transfection, respectively. A 10-µl sterile pipette tip was applied to scratch the cells. Three wounds were made for each group. The scratched wound was photographed and the distance was measured every 24 hr. Invasion assay was conducted by transwell insert chambers with Matrigel (BD Biosciences). 2 × 10^4^ transfected cells were seeded into the upper Biocoat with 200 μl serum-free DMEM. The lower biocoat was supplemented with 500 μl 10% FBS DMEM to create a chemokine-induced environment. After 48 h, the cells were fixed with 4% paraformaldehyde for 30 min and stained with crystal violet 0.5% for 10 min. Then five random visual fields were photographed.

### Cell cycle and apoptosis assays

Cells were washed twice with PBS 48 h after transfection and then stained with 100 µl propidium iodide (PI) for 15 min at 4 °C in dark. The cell cycle assays were detected by flow cytometry with a flow cytometer (FACS Calibur^TM^, BD Biosciences, CA, USA). The results were analyzed using the ModFit LT software.

Apoptosis assay and flow cytometry were performed using the Annexin V-fluorescein isothiocynate Apoptosis Detection Kit (KeyGen, Nanjing, China) according to the manufacturer’s guidelines. The number of early apoptotic cells and late apoptotic cells were considered as total apoptotic cells.

### Western blotting

Cells were harvested 48 h after transfection. Samples were probed with primary antibody (Supplementary Table 6). Goat anti-rabbit or anti-mouse HRP antibodies were used (ZhongshanJinqiao Company, Beijing, China). ECL detection system (Millipore, Bedford, MA, USA) was applied to determine protein expression. The antibodies used in this study were provided in Supplementary Table 6.

### Immunohistochemical (IHC) staining

IHC staining was performed according to our previous study^41^. Briefly, sections were deparaffinized and rehydrated, then stained in Mayer hematoxylin solution for 8 min. Sections were washed in warm running tap water for 10 min, rinsed in distilled water and 95% alcohol for 10 dips. Counterstain in eosin-phloxine Y solution for 1 min. Dehydrate sections through 95% alcohol, 2 times of absolute alcohol, 5 min each time. Mount sections with xylene based mounting medium. For DDX11, p-PI3K, p-AKT, mTOR and Ki67 staining, antigen-retrieved sections were washed 2 times with PBS and 1 time with PBST. After blocking at room temperature with 3% BSA for 1 h, slides were incubated with antibodies for 1 h at room temperature. The expression was determined with the HRP-DAB system (Millipore, USA). The antibodies used in this study were provided in Supplementary Table 6.

### Luciferase activity assay

The human DDX11 promoter fragment was cloned into a pGL3 vector to perform luciferase activity assays. Cells were seed in 24 well plates and transfected with E2F1 plasmids. After 48 h, cells were collected and lysed for luciferase assays. The relative luciferase activity was calculated by normalizing the Firefly luminescence to the Renilla luminescence as our previous study^42^.

### Chromatin immunoprecipitation (ChIP) assay

The ChIP assay was conducted with the EZ ChIP™ Chromatin Immunoprecipitation Kit (Millipore, USA) according to the manufacturer’s protocol. E2F1 antibody and IgG control antibody were purchased from Proteintech Company (Wuhan, China). The enriched DNA was analyzed by real-time PCR.

### Animal experiments

6–8-weeks old male mice (6 weeks old) were purchased from Beijing Vital River Laboratory Animal Technology Co., Ltd, China and kept in a controlled environment (12 hr light–dark cycle, 25 °C, and 60–70% humidity) in the First Affiliated Hospital of Zhengzhou University. Mice were used for the subcutaneous tumor formation assay. Briefly, 1 × 10^7^ stable DDX11 suppression cells (lenti-sh-DDX11) and negative control (Lenti-MOCK), as well as stable DDX11 overexpression cells (oe-DDX11) and negative control (oe-NC) were subcutaneously (s.c.) injected into the lower flank of the mice. Tumor volume was determined with the formula: Volume = (width)^2^ × length/2. Mice were pictured with IVIS Lumina II system (Caliper Life Sciences, Hopkinton, MA) as described in our previous study^42^. Tumors were surgically removed and weighed after around 5 weeks. The studies were approved by the Experimental Animal Ethics Committee of The First Affiliated Hospital of Zhengzhou University.

### Statistical analysis

All the statistical analyses were performed using the SPSS 20.0 software program (SPSS Inc., Chicago, USA). The Student’s *t*-test was conducted to address the difference between two groups of data. Clinico-pathological variables were analyzed by chi-square tests. Kaplan–Meier curves and the log-rank tests were used to analyze the overall survival (OS) or disease-free survival (DFS) of HCC patients. Cox regression analysis of univariate and multivariate was performed to ascertain independent factors. *P* < 0.05 is considered to be statistically significant. All data were presented as mean ± SD. All experiments were performed at least three times.

## Supplementary information

Supplementary Figure Legend

Supplementary Figure 1

Supplementary Figure 2

Supplementary Figure 3

Supplementary Figure 4

Supplementary Figure 5

Supplementary Figure 6

Supplementary Figure 7

Supplementary Figure 8

Supplementary Figure 9

Supplementary Table 1-6
