# Supplementary material for: E2F1 mediated DDX11 transcriptional activation promotes hepatocellular carcinoma progression through PI3K/AKT/mTOR pathway
Source: Cell Death Dis. 2020 Apr 24;11(4):273. doi: 10.1038/s41419-020-2478-0 (PMC7181644; doi:10.1038/s41419-020-2478-0)
Supplement: Supplementary file 1 — Supplementary Figure Legend [file 41419_2020_2478_MOESM1_ESM.docx]

Supplementary Figures

Figure S1. Immunohistochemistry staining of DDX11 in pan-cancer and ZZU HCC tissue microarray (TMA). (A-C) IHC staining of DDX11 expression in pan-cancer tissues and paired non-tumor tissues. (D-J) IHC staining of DDX11 expression in ZZU HCC cohort containing HCC tissues and adjacent non-tumor tissues.

**Figure S2. Kaplan-Meier analysis of overall survival (OS)/disease-free survival (DFS) in Pan-cancers with high or low DDX11 expression based on TCGA database.**

**Figure S3. The Gene Set Enrichment Analysis (GSEA) plot analysis of the correlation between DDX11 expression and the genes expressed in hepatocellular carcinomas with a poor survival prognosis or high recurrence rate**.

**Figure S4. The correlation between DDX11 expression and Ki67, AFP expression and DDX11 promotes cell migration in HCC cells. (A)** Bioinformatics analysis of the association between DDX11 and Ki67, or AFP expression. **(B)** Bioinformatics analysis of DDX11 expression in normal liver tissue, cirrhotic tissue, low-grade dysplastic tissue, high-grade dysplastic tissue, early HCC and advanced HCC tissues in GSE6764 dataset. **(C)** The ROC curve analysis indicated DDX11 could be a potential diagnostic biomarker for HCC patients. **(D)** The cell migration ability of HepG2 or SMMC7721 cells transfected with sh-NC/sh-DDX11 was assessed by wound-healing assay. **p* < 0.05, ***p* < 0.01.

**Figure S5. Overexpression of DDX11 promotes cell proliferation, migration and invasion, and inhibits apoptosis of HCC cells *in vitro*.
(A)** Western blot and qPCR analysis of DDX11 expression in Hep3B or Huh7 cells after transfection of DDX11 overexpression plasmid (oe-DDX11) or negative control (oe-NC). Hep3B or Huh7 cells were transfected with oe-NC or oe-DDX11. **(B-D)** Cell proliferation and DNA synthesis were analyzed by CCK-8 assay, colony formation assay and EdU immunofluorescence staining respectively. **(E)** Cell invasion ability was determined by transwell assay. **(F)** Cell migration capability was assessed by wound-healing assay. **(G-H)** Cell apoptosis and cell cycle analysis of Hep3B or Huh7 cells transfected with oe-NC or oe-DDX11 were analyzed by flow cytometer using Annexin V/PI or PI staining. * *p* < 0.05, ***p* < 0.01, ****p* < 0.001.

**Figure S6. Bioinformatics analysis of DDX11 involved biological processes and signaling pathways based on TCGA-LIHC cohort dataset. (A)** Differentially expressed genes were analyzed based on DDX11 expression in TCGA-LIHC cohort. **(B-D)** Gene ontology (GO) and Kyoto Encyclopedia of Genes and Genomes (KEGG) pathway enrichment analysis was performed based on DDX11 expression in TCGA-LIHC cohort. **(E)** The Gene Set Enrichment Analysis (GSEA) plot analysis of the correlation between DDX11 expression and cell cycle, DNA replication, homologous recombination, or mismatch repair.

**Fig. S7 Exploration of DDX11 action pathway. (A-B)** Cell colony formation (up panel) and invasion (lower panel) of SMMC7721 cells transfected with DDX11 siRNA or Negative control, DDX11 plasmid or negative control, or cultured with DMSO or LY294002. The representative result of at least three independent experiments was shown. Scale bars, 8mm. Invasion: Scale bars, 50μm

**Figure S8. The correlation between DDX11 and E2F1 expressions in pan-cancer from TCGA database.**

**Abbreviations:** Adrenocortical carcinoma (ACC), Bladder urothelial carcinoma (BLCA), Breast cancer (BRCA), Cholangiocarcinoma (CHOL), Colon cancer (COAD), DLBC (Lymphoid Neoplasm Diffuse Large B-cell Lymphoma), Esophagus cancer (ESCA), Glioblastoma multiforme (GBM), Head and Neck squamous cell carcinoma (HNSC), Kidney renal clear cell carcinoma (KIRC), Kidney renal papillary cell carcinoma (KIRP), Brain Lower Grade Glioma (LGG), Liver cancer (LIHC), Lung cancer (LUAD), Lung squamous cell carcinoma (LUSC), Ovarian serous cystadenocarcinoma (OV), Pancreatic adenocarcinoma (PAAD), Pheochromocytoma and Paraganglioma (PCPG), Prostate adenocarcinoma (PRAD), Rectal cancer (READ), Sarcoma (SARC), Skin Cutaneous Melanoma (SKCM), Stomach cancer (STAD), Testicular Germ Cell Tumors (TGCT), Thyroid cancer (THCA), Thymoma (THYM) and Uterine Carcinosarcoma (UCS), Acute Myeloid Leukemia (LAML).

**Figure S9. E2F1-DDX11 correlates with HCC patient outcomes**. Kaplan-Meier analysis of the overall survival (A) or recurrence-free survival (B) in HCC patients according to the concurrent expression of E2F1 and DDX11.
