# Supplementary figures and images for: E2F1 mediated DDX11 transcriptional activation promotes hepatocellular carcinoma progression through PI3K/AKT/mTOR pathway
Source: Cell Death Dis. 2020 Apr 24;11(4):273. doi: 10.1038/s41419-020-2478-0 (PMC7181644; doi:10.1038/s41419-020-2478-0)

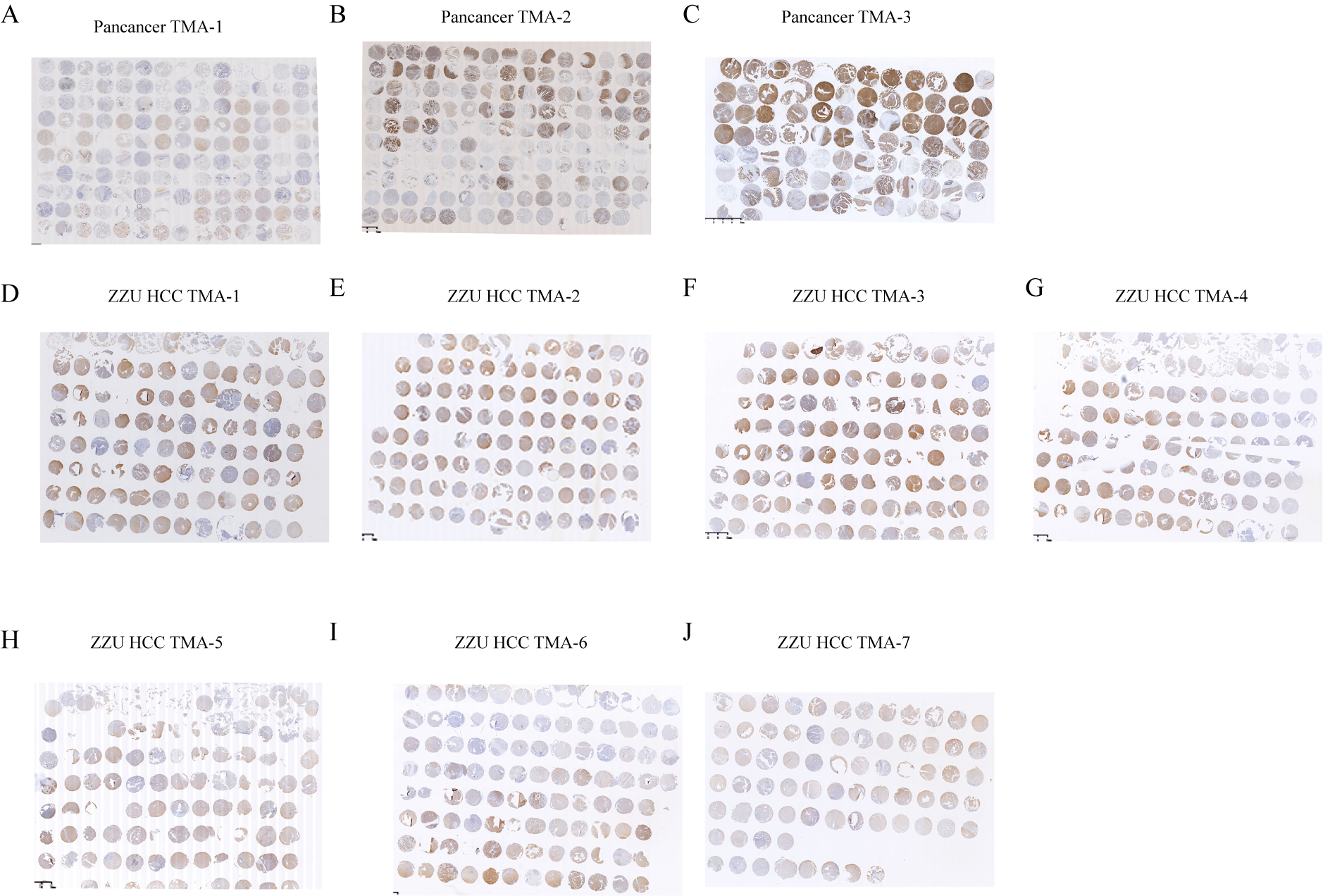

Supplement: Supplementary file 2 — Supplementary Figure 1 [file 41419_2020_2478_MOESM2_ESM.tif]

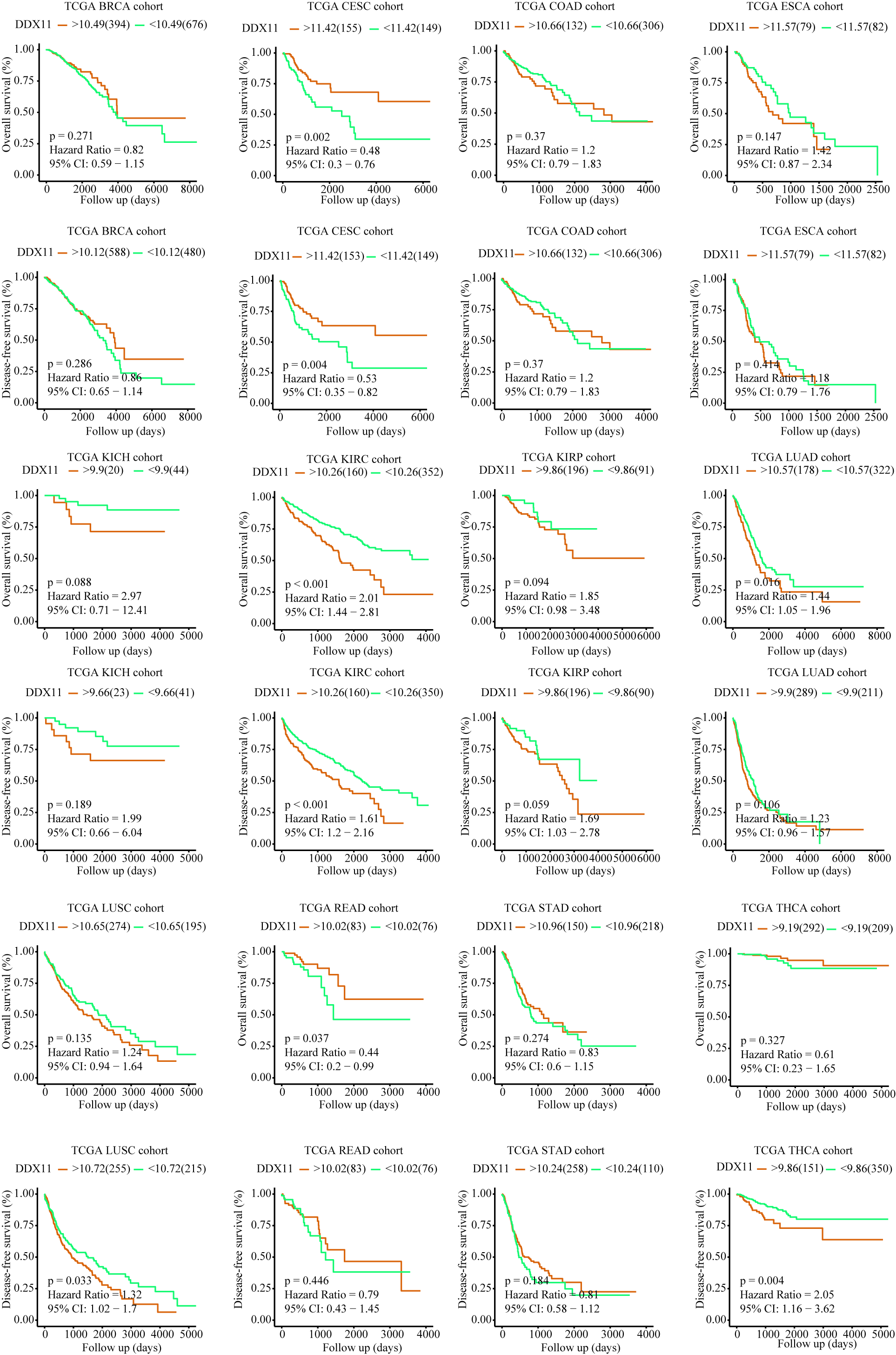

Supplement: Supplementary file 3 — Supplementary Figure 2 [file 41419_2020_2478_MOESM3_ESM.tif]

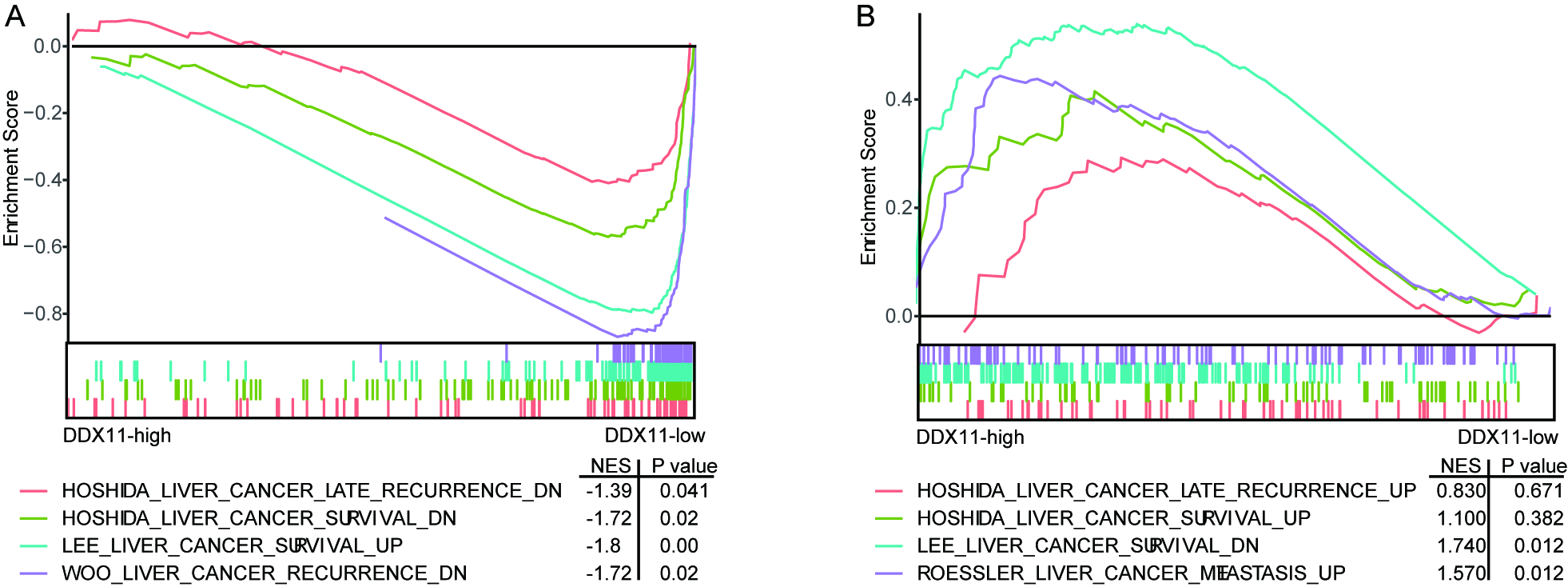

Supplement: Supplementary file 4 — Supplementary Figure 3 [file 41419_2020_2478_MOESM4_ESM.tif]

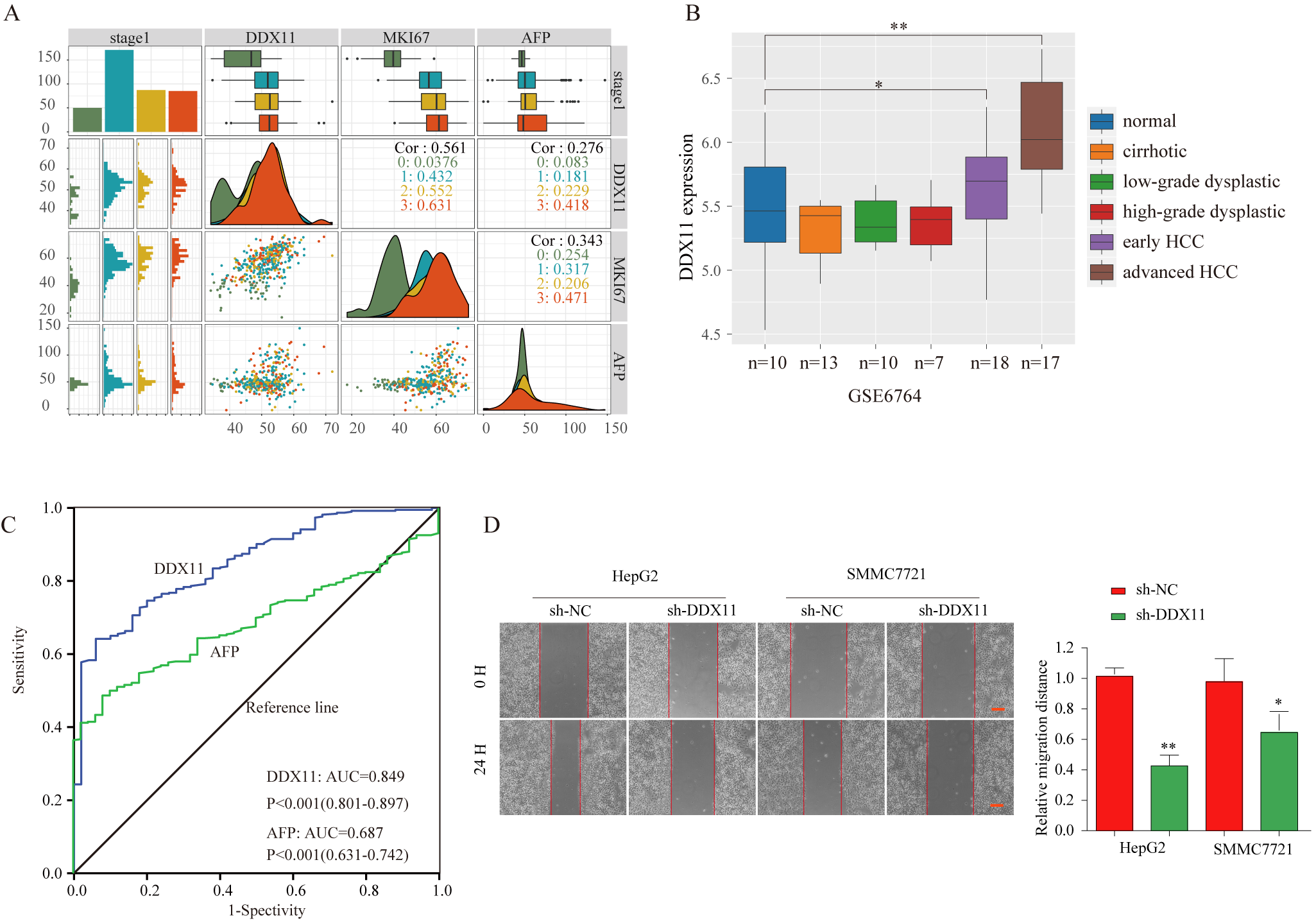

Supplement: Supplementary file 5 — Supplementary Figure 4 [file 41419_2020_2478_MOESM5_ESM.tif]

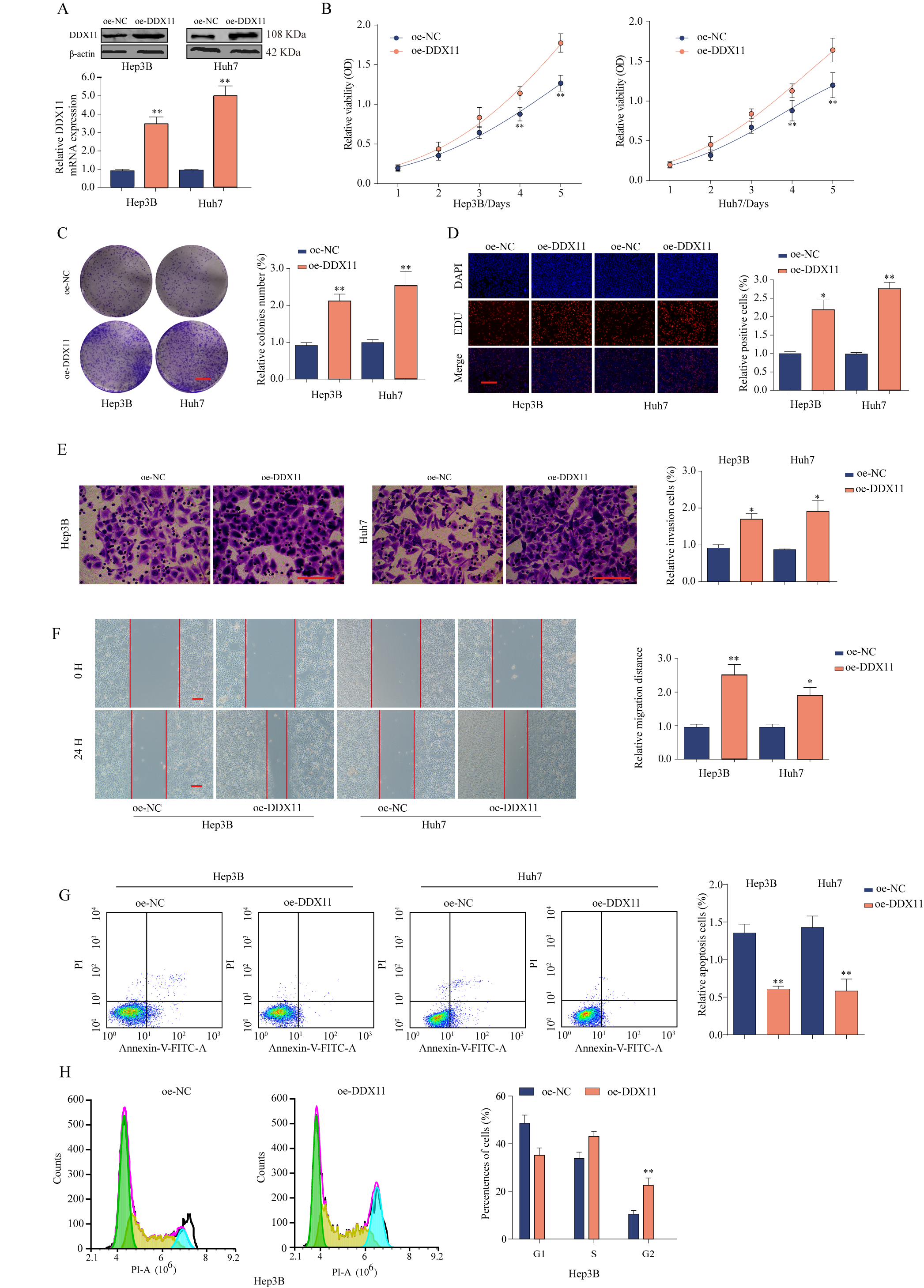

Supplement: Supplementary file 6 — Supplementary Figure 5 [file 41419_2020_2478_MOESM6_ESM.tif]

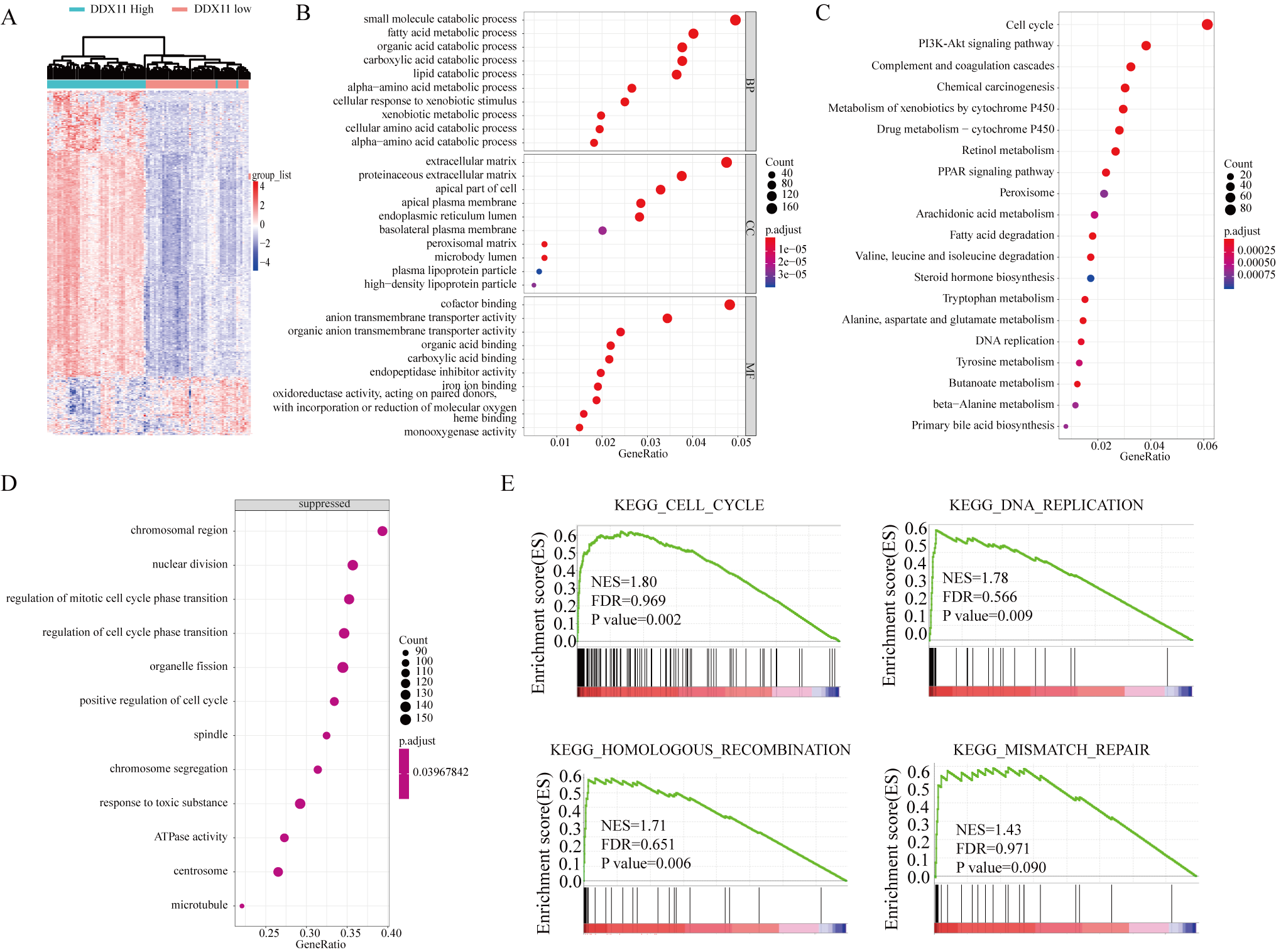

Supplement: Supplementary file 7 — Supplementary Figure 6 [file 41419_2020_2478_MOESM7_ESM.tif]

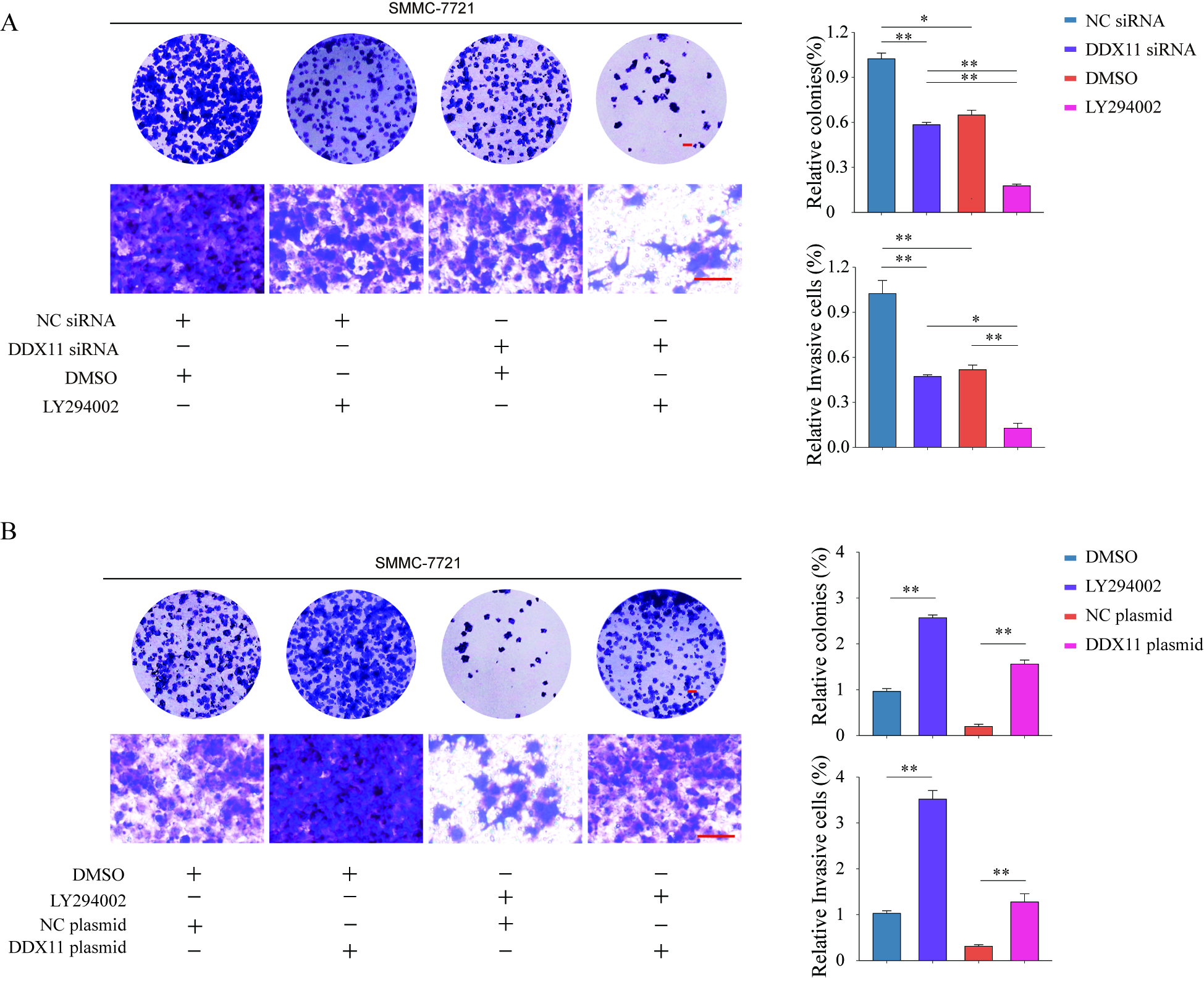

Supplement: Supplementary file 8 — Supplementary Figure 7 [file 41419_2020_2478_MOESM8_ESM.tif]

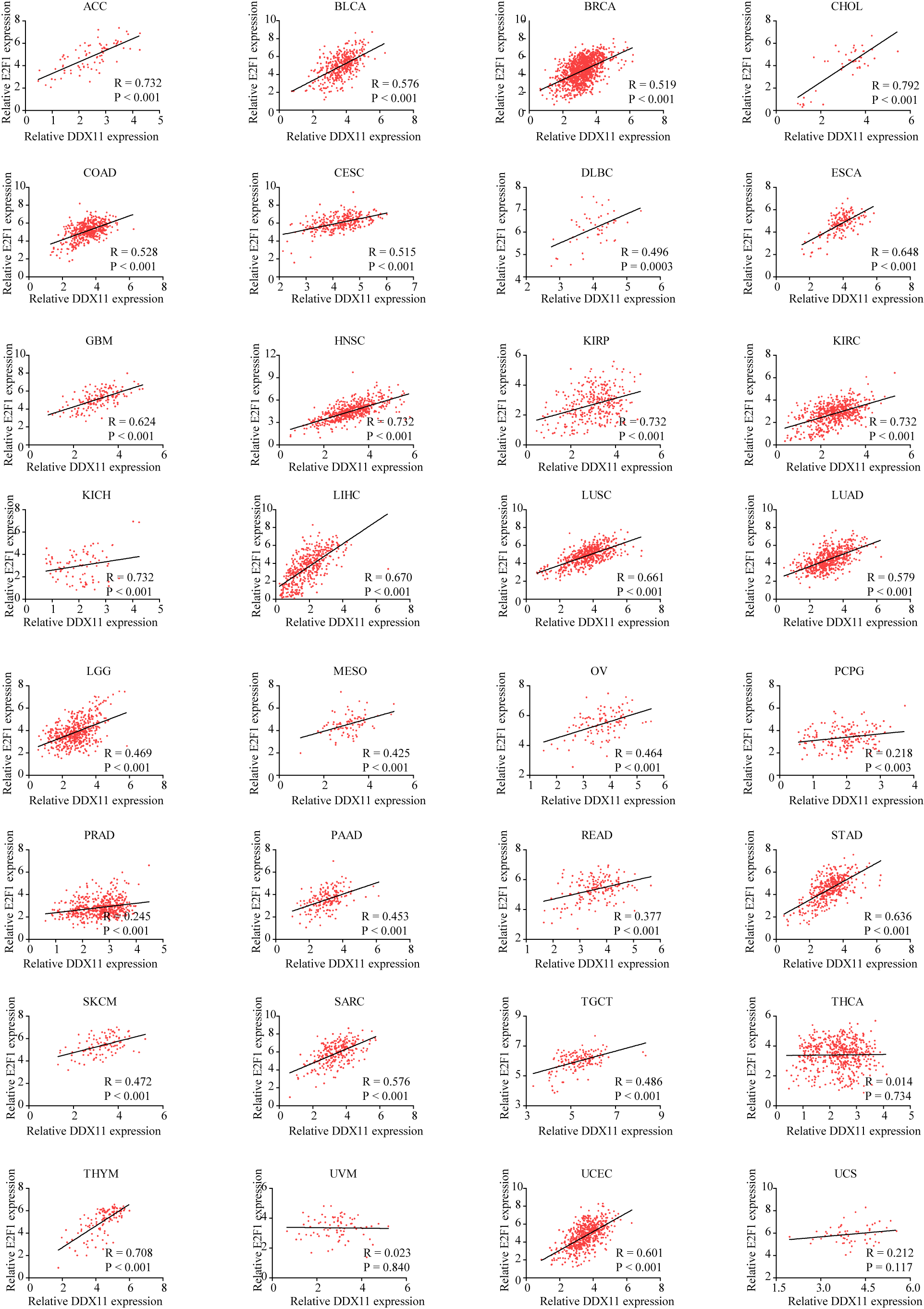

Supplement: Supplementary file 9 — Supplementary Figure 8 [file 41419_2020_2478_MOESM9_ESM.tif]

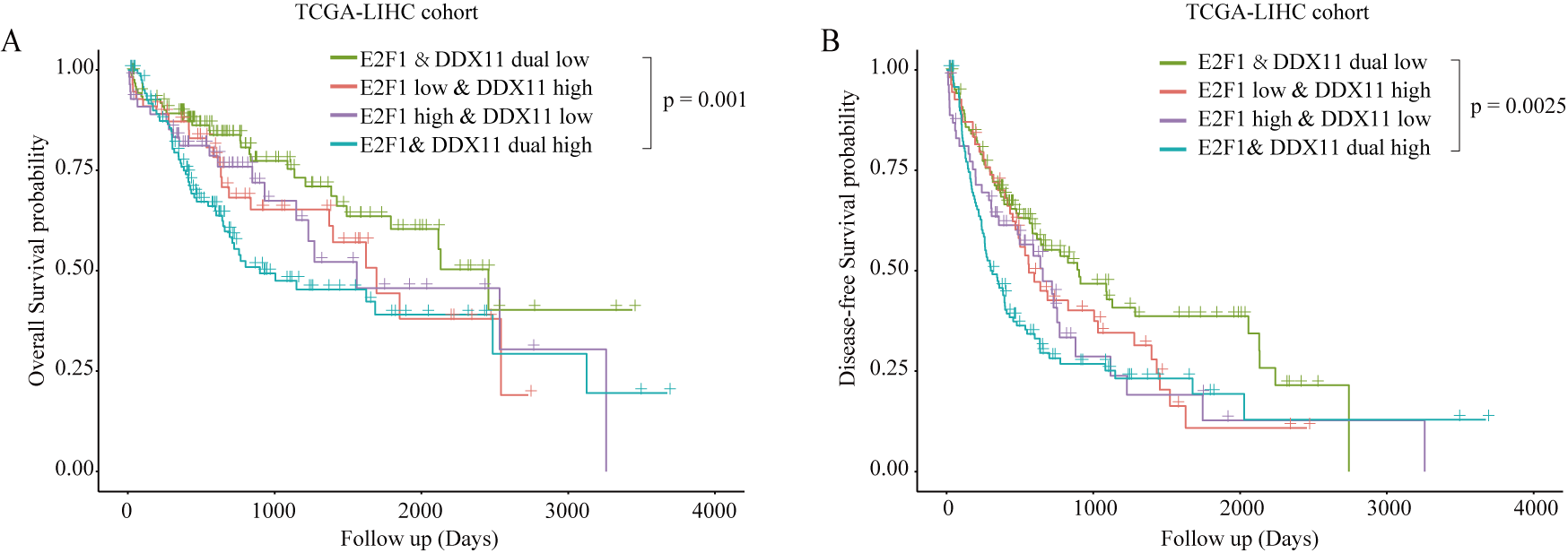

Supplement: Supplementary file 10 — Supplementary Figure 9 [file 41419_2020_2478_MOESM10_ESM.tif]
