# Supplementary material for: E2F1 mediated DDX11 transcriptional activation promotes hepatocellular carcinoma progression through PI3K/AKT/mTOR pathway
Source: Cell Death Dis. 2020 Apr 24;11(4):273. doi: 10.1038/s41419-020-2478-0 (PMC7181644; doi:10.1038/s41419-020-2478-0)
Supplement: Supplementary file 11 — Supplementary Table 1-6 [file 41419_2020_2478_MOESM11_ESM.docx]

**Supplementary Table 1. Association of DDX11 expression and clinical features in TCGA cohort**

| Variables | Clinicopathological features | DDX11 | | P-value |
| --- | --- | --- | --- | --- |
|  |  | Low (n=175) | High (n=170) |  |
| Age (years) | ≤median | 89 | 82 | 0.626 |
|  | >median | 86 | 88 |  |
| Gender | Male | 51 | 59 | 0.268 |
|  | Female | 124 | 111 |  |
| TNM stage | Stage I and II | 135 | 120 | 0.166 |
|  | Stage III and IV | 40 | 50 |  |
| Differentiation grade | Grade I and II | 123 | 92 | **0.002** |
|  | Grade III and IV | 52 | 78 |  |
| Serum AFP(ng/mL) | ≤200 | 74 | 44 | **0.001** |
|  | >200 | 101 | 126 |  |

Bold values indicate statistical significance, *P* < 0.05

**Supplementary Table 2. Univariate and multivariate analyses of overall survival of HCC**

| Univariate analyses | Clinicopathological parameters | Overall survival (OS) | | | Disease-free survival (DFS) | | |
| --- | --- | --- | --- | --- | --- | --- | --- |
|  |  | HR | 95%（CI） | *P* value | HR | 95%（CI） | *P* value |
|  | Age (>median vs. ≤median) | 1.251 | 0.863-1.813 | 0.237 | 1.070 | 0.812-1.410 | 0.633 |
|  | Gender (male vs. female) | 1.300 | 0.891-1.897 | 0.174 | 1.166 | 0.873-1.557 | 0.298 |
|  | TNM stage (III-IV vs. I-II) | 2.516 | 1.732-3.656 | **<0.001** | 2.075 | 1.543-2.790 | **<0.001** |
|  | Differentiation grade ( III-IV vs. I-II ) | 1.151 | 0.791-1.675 | 0.462 | 1.110 | 0.838-1.472 | 0.467 |
|  | AFP expression ( High vs. Low) | 1.039 | 0.707-1.528 | 0.845 | 1.001 | 0.751-1.333 | 0.996 |
|  | DDX11 expression (High vs. Low) | 1.655 | 1.141-2.401 | **0.008** | 1.648 | 1.248-2.176 | **<0.001** |
| Multivariate analyses | TNM stage (III-IV vs. I-II) | 2.454 | 1.688-3.568 | **<0.001** | 2.023 | 1.503-2.724 | **<0.001** |
|  | DDX11 expression (High vs. Low) | 1.588 | 1.093-2.306 | **0.015** | 1.600 | 1.211-2.115 | **0.001** |

Bold values indicate statistical significance, *P* < 0.05

**Supplementary Table 3.** Cell lines used in this study

| **Cell lines** | **Type** | **Sourse** |
| --- | --- | --- |
| LO2 | Normal liver cell | Sibcb, China |
| HepG2 | Liver cancer cell | ATCC,USA |
| SMMC7721 | Liver cancer cell | Sibcb, China |
| Hep3B | Liver cancer cell | Sibcb, China |
| Huh7 | Liver cancer cell | Sibcb, China |

| Cohort ID | Platform | Number of samples | | Public year | Country |
| --- | --- | --- | --- | --- | --- |
|  |  | Non-tumor | Tumor |  |  |
| TCGA | Illumina | 50 | 374 | 2009 | USA |
| GSE6764 | Affymetrix | 40 | 35 | 2007 | USA |
| GSE10143 | DASL | 307 | 80 | 2008 | USA |
| GSE22058 | Affymetrix | 220 | 225 | 2010 | USA |
| GSE39791 | Illumina | 72 | 72 | 2014 | USA |
| GSE45436 | Affymetrix | 41 | 93 | 2014 | Taiwan China |
| GSE64041 | Affymetrix | 60 | 60 | 2016 | Switzerland |
| GSE102083 | Affymetrix | 105 | 152 | 2018 | Japan |
| Total |  | **895** | **1091** |  |  |

**Supplementary Table 4. HCC expression profile cohorts used in this study**

**Supplementary Table 5.** Primer sequence used in this study

| Name | Direction | Primer(5’-3’) |
| --- | --- | --- |
| DDX11 | Forward | 5'- GCAAGGATGTTCGGCTGGTCTC-3' |
|  | Reverse | 5'- CTTCTTCTCGTGCCTGCTTCTCTG-3' |
| E2F1 | Forward | 5'- AGGCTGGATCTGGAGACTGA-3' |
|  | Reverse | 5'- CTTCAAGCCGCTTACCAATC-3' |
| β-actin | Forward | 5'- CAGGGCGTGATGGTGGGCA-3' |
|  | Reverse | 5'- CAAACATCATCTGGGTCATCTTCTC-3' |

**Supplementary Table 6.** Information on antibodies used in present study.

| Antibody | WB | IHC | Specificity | Company |
| --- | --- | --- | --- | --- |
| β-actin | 1:2000 | / | Mouse monoclonal | Sigma.USA |
| DDX11 | 1:1000 | 1:100 | Mouse monoclonal | Millipore, USA |
| p-PI3K | 1:1000 | 1:100 | Mouse monoclonal | Millipore, USA |
| p-AKT | 1:1000 | 1:100 | Mouse monoclonal | Millipore, USA |
| mTOR | 1:1000 | 1:100 | Mouse monoclonal | Millipore, USA |
| Ki-67 | / | 1:100 | Mouse monoclonal | Millipore, USA |
| E2F1 | 1:1000 | / | Rabbit monoclonal | Proteintech, China |
